# Rocaglates and pateamines target the DEAD-box RNA helicase eIF4A from *Schistosoma mansoni* and demonstrate anti-schistosomal activity *in vitro*

**DOI:** 10.1038/s41598-026-63203-w

**Published:** 2026-08-01

**Authors:** Sophie Welsch, Francesca Magari, Annika S. Mokosch, Simone Haeberlein, Stefanie Gerbig, Bernhard Spengler, Arnold Grünweller, Christoph G. Grevelding

**Affiliations:** 1 Biomedical Research Center Seltersberg (BFS), Institute of Parasitology, Justus Liebig University Giessen, 35392 Giessen, Germany; 2 Institute of Pharmaceutical Chemistry, Philipps University Marburg, 35037 Marburg, Germany; 3 Institute of Inorganic and Analytical Chemistry, Justus Liebig University Giessen, 35392 Giessen, Germany; 4 TransMIT Gesellschaft für Technologietransfer mbH, 35394 Giessen, Germany

**Keywords:** *Schistosoma mansoni*, Rocaglates, Pateamines, eIF4A, Translation initiation, Stem cells, Biochemistry, Drug discovery, Microbiology

## Abstract

For decades, infections with the parasite *Schistosoma mansoni* have been treated with praziquantel, the only available drug, which highlights the urgent need of treatment alternatives. Natural compounds including rocaglates from plants of the genus *Aglaia* and pateamines isolated from the marine sponge *Mycale hentscheli* have exhibited promising anti-viral, anti-cancer, and anti-pathogenic properties. These compounds target the eukaryotic translation initiation factor 4A (eIF4A), which is a crucial factor for unwinding RNA structures in the 5’-UTR of selected mRNAs, and prevent the unwinding activity of eIF4A, which interferes with protein synthesis. Preliminary data indicated anthelminthic activity of the rocaglate silvestrol against *S. mansoni in vitro.* In this study, we confirmed the interaction of rocaglates and pateamines with recombinant *S. mansoni* eIF4A and analyzed the activity of these compounds against adult *S. mansoni in vitro.* Our findings showed reduced parasite vitality and stem-cell proliferation as well as impaired embryogenesis after rocaglate or pateamine treatment in the nanomolar range. Furthermore, we observed that rocaglate-treated *S. mansoni* recovered during washout experiments, while pateamine treatment displayed irreversible effects finally killing most of the worms. Although some properties of the used compounds impede their suitability as anti-schistosomal candidates, eIF4A remains a promising drug target in this parasite.

**Supplementary Information:**

The online version contains supplementary material available at 10.1038/s41598-026-63203-w.

## Introduction

The targeted manipulation of mRNA translation with drugs has proven to be a potential therapeutic strategy for diseases that are characterized by malfunctions of gene regulation such as cancer^1,2^. In this context, the eukaryotic translation initiation factor 4A (eIF4A) came to the fore^3^. eIF4A is part of the heterotrimeric eIF4F complex and, as an RNA helicase, it unwinds stable secondary structures in the 5’-untranslated region (5’-UTR) of selected mRNAs^4–7^. Several eIF4A inhibitors have been identified, including the compound classes rocaglates and pateamines, which are derived from natural sources. Rocaglates are isolated from *Aglaia* plants, while pateamine A (PatA) is extracted from the marine sponge *Mycale hentscheli*^8^. These compounds inhibit eIF4A by clamping RNA substrates on the protein surface and thus locking eIF4A in this ternary complex^9–11^. Six amino acids in the eIF4A:RNA:rocaglate binding pocket were found to be detrimental for this clamping mechanism: Thr158, Pro159, Phe163, Phe192, Gln195, and Ile199 (positions refer to human eIF4A)^10,12,13^. *In silico* analysis of the amino acid at position 163 was shown to be useful for predicting rocaglate sensitivity or non-sensitivity in various organisms. While either Phe, Tyr, His, or Val at this position were described as rocaglate sensitive, the presence of Leu, Ile, Ser or Gly resulted in rocaglate non-sensitivity^10,13,14^. In contrast to rocaglates, the clamping mechanism of pateamines is independent of the amino acid at position 163^15^. Inhibition of eIF4A by rocaglates or pateamines showed anti-proliferative effects *in vitro* and *in vivo*, including clinical trials^16–19^. Furthermore, anti-viral properties against a variety of viruses^15,20,21^, as well as anti-pathogenic potential against *Candida auris*^22^ have been described. Finally, the anti-parasitic potential of eIF4A inhibitors was also demonstrated for protozoan parasites like *Plasmodium falciparum*^23^, *Leishmania major*^15^, and *Toxoplasma gondii*^14^, and preliminary evidence was obtained for effects on the multicellular parasite *Schistosoma mansoni*^14^.

*S. mansoni* and further schistosome species infect humans and animals, causing schistosomiasis, one of the most important neglected tropical diseases^24–26^. Estimates of the World Health Organization showed that more than 250 million people are infected and require treatment^27^. Although schistosomiasis is mainly prevalent in tropical and subtropical regions^28^, cases have also been reported in Corsica^29^ and Spain^30^, indicating the risk of transmission in southern Europe^31^.

The complex life cycle of *S. mansoni* includes the freshwater snail *Biomphalaria glabrata* as an intermediate and vertebrates as final hosts. When excreted eggs enter freshwater, miracidia (first free-living larval stage) hatch from the eggs and infect *B. glabrata* snails. Inside the snail, miracidia develop through several sporocyst stages into cercariae (second free-living larval stage), which are released into the water. As infectious stage for humans and animals, cercariae penetrate the skin of their final hosts, develop into schistosomula, and circulate in the bloodstream until they reach the liver. Here, schistosomula mature into adult worms of separate sexes, which pair and migrate as couples to the mesenteric veins of the intestine as their final destination^24^. Pairing induces the sexual maturation of the female by inducing processes that lead to the final differentiation of the reproductive organs and finally to egg production. Couples produce up to 300 eggs per day, which enter the gut lumen and are excreted with the feces to reach the environment and complete the parasite’s life cycle. Some of the eggs are not excreted but remain in the human (or animal) body and are trapped in organs like liver or spleen. These eggs induce inflammatory processes that lead to liver fibrosis. Therefore, the eggs are the pathogenic factor of this disease^28^. To date, no effective vaccine is available. The control of schistosomiasis still relies on massive drug administration of praziquantel (PZQ), which is the only available drug^32^. PZQ has been used to treat schistosomiasis since the 1970s. Meanwhile, reduced susceptibility in the field and resistance in laboratories have been documented^33–35^, emphasizing the urgent need of new targets and drugs.

Schistosomiasis has been described as a stem-cell driven disease^36^, because stem-cell activity of the parasites’ germinal stem cells (GSCs) is necessary for reproduction^37,38^ and that of somatic stem cells (SSCs; neoblasts) rejuvenates tissues like the tegument, parenchyma, and gut to enable parasite survival in the host’s bloodstream^39,40^. Since life-cycle maintenance including growth to the adult stage requires cell proliferation and differentiation^37^, it is likely that schistosome eIF4A helicases fulfill similar roles as their orthologs from model organisms and parasitic protozoans, for which these roles have already been described^41–44^. Indeed, our recent molecular characterization of two RNA helicases demonstrated the importance of *S. mansoni* eIF4A-a (SmeIF4A-a) for stem-cell proliferation, gonad function, and egg production^45^. For these reasons, we considered SmeIF4A-a as a potential drug target in this parasite. This assumption was substantiated by first results with the natural rocaglate silvestrol, which showed reduced motility and egg production in adult *S. mansoni in vitro*^14^.

Here, we analyzed the antiparasitic potential of rocaglates and pateamines against adult *S. mansoni in vitro* and investigated the interactions of these eIF4A inhibitors and SmeIF4A isoforms. The results demonstrated anti-schistosomal activity of both compound classes and their roles in inhibiting cell proliferation and parasite development.

## Results

### Thermal denaturation measurements confirmed SmeIF4A-a sensitivity towards rocaglates and pateamines

**Fig. 1:**
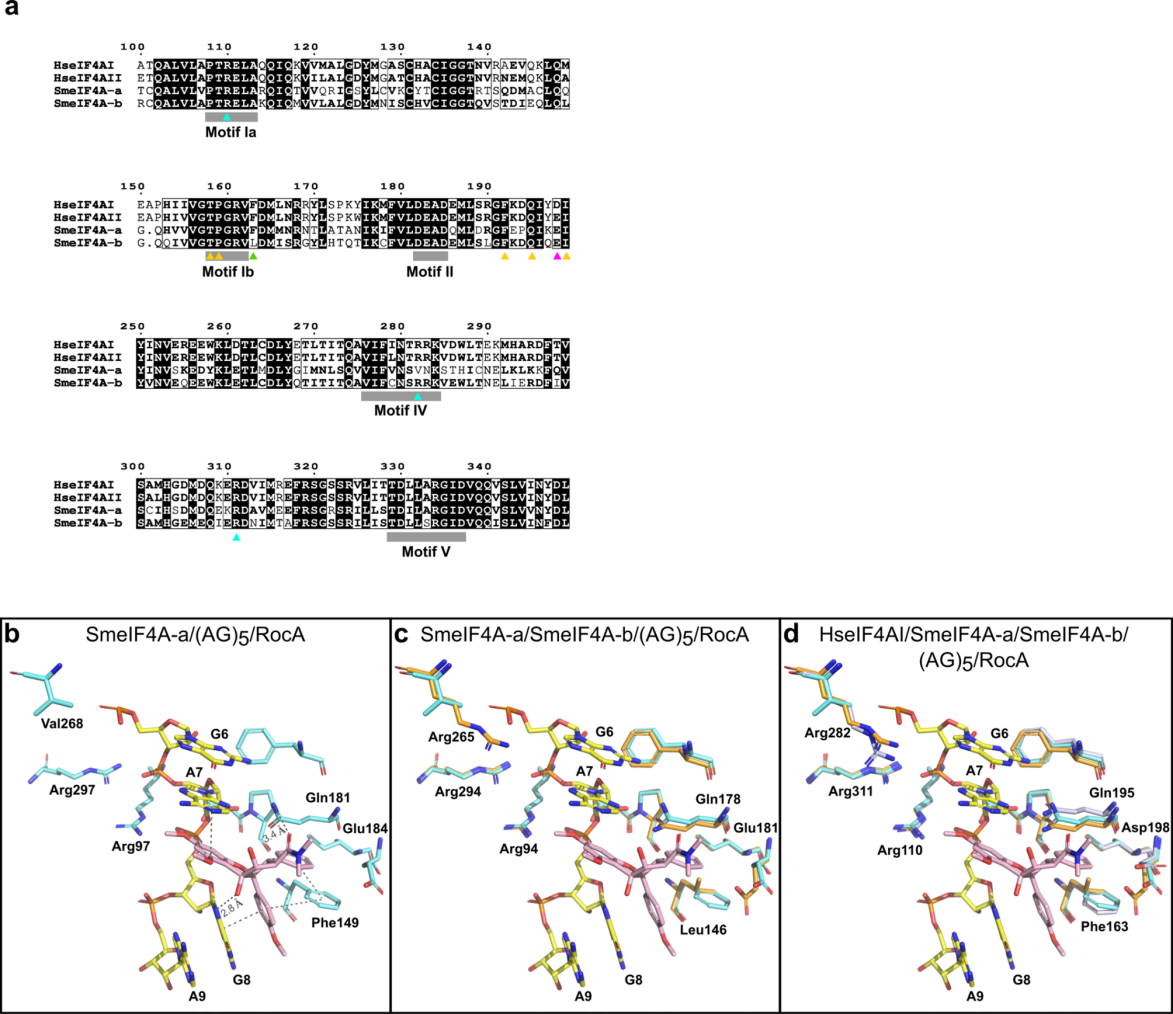
SmeIF4A-a but not SmeIF4A-b was predicted to be rocaglate sensitive. **(a)** A multiple sequence alignment of both human (HseIF4AI, HseIF4AII) and *S. mansoni* eIF4A isoforms showed five of the nine conserved motifs (grey bars) typical for DEAD-box RNA helicases^97^ as well as additional aa involved in rocaglate or pateamine binding (arrowheads)^14,15^. Six aa are present in the RNA/rocaglate binding pocket (yellow arrowheads) and one of them, the aa at position 163 of HseIF4A (green arrowhead), predicted rocaglate sensitivity or non-sensitivity^14^. SmeIF4A-a has the same binding motif (TPFFQI) as human eIF4A, while SmeIF4A-b contains a Leu at the third position (TPLFQI). The dioxane moiety of silvestrol interacts with three Arg residues (cyan arrowheads)^14^. One of these residues is replaced by Val in SmeIF4A-a, while all Arg residues are present in the other proteins shown. The aa Asp198 (magenta arrowhead) may form a hydrogen bond with PatA^15^, but it is only present in HseIF4AI, while all other proteins contain a Glu. **(b)** The rocaglate RocA (pink stick model, PDB: 5ZC9) in complex with the polypurine RNA (AG)_5_ (yellow sticks with phosphorus atoms colored orange) binds in the RNA binding pocket of SmeIF4A-a (b, c, and d, cyan stick - nitrogen atoms in blue, oxygen atoms in red), SmeIF4A-b (c and d, orange stick), and human eIF4A (d, silver stick for comparison). RocA is involved in two hydrogen bonds with Gln181 (human Gln195 in c) and RNA G8. Furthermore, it binds via several π-π stacking interactions the RNA A7, G8, and the Phe149 (human Phe163 in c), which play an important role in the clamping mechanism of rocaglates. **(c)** In SmeIF4A-b, the Phe is replaced with a Leu (Leu146). **(d)** The typical human arginine pocket (Arg110, Arg282 and Arg311), which is important for the eIF4A-RNA complex formation prior to rocaglates clamping^14^, corresponds to SmeIF4A-a Arg97, Val268, and Arg297 (b) and to SmeIF4A-b Arg94, Arg265, and Arg294 (c).

Two human eIF4A isoforms (HseIF4AI, HseIF4AII) are part of the eIF4F complex and involved in translation initiation^46–48^. Sensitivity or non-sensitivity of these eIF4A isoforms to rocaglates was previously predicted by the amino acid present at position 163 in the RNA/rocaglate binding pocket. In *S. mansoni*, the two eIF4A isoforms SmeIF4A-a and SmeIF4A-b were discovered by * in silico* analysis and characterized at the molecular level. SmeIF4A-a is 57 % identical to human eIF4A, while SmeIF4A-b shares an amino acid sequence identity of about 70 % to human eIF4A^45^. The sequence of the rocaglate-binding motif as well as the presence of additional amino acids involved in rocaglate or pateamine binding were investigated by multiple sequence alignments of the two human and *S. mansoni* eIF4A isoforms. SmeIF4A-a contains the same rocaglate binding motif (TPFFQI) as human eIF4A, while SmeIF4A-b has a Leu instead of Phe at the third position (TPLFQI). The Arg-rich pocket of human eIF4A, which contains Arg110, Arg282, and Arg311, interacts with RNA and the dioxane moiety of silvestrol. Based on mutation and docking studies, the importance of each Arg for complex formation and silvestrol binding was predicted in the following order: Arg311 > Arg110 > Arg282^14^. SmeIF4A-a has a Val268 instead of Arg282, while SmeIF4A-b contains all three Arg residues (Fig. 1). Former docking analysis of human eIF4A and PatA indicated that their interaction includes an additional hydrogen bond at Asp198^15^. This amino acid is only present in HseIF4AI, while HseIF4AII, and both *S. mansoni* eIF4A isoforms have a Glu at that position (Fig. 1).

For docking studies, AlphaFold3-predicted^49^ models of SmeIF4A-a and SmeIF4A-b, respectively, were employed since no experimentally determined structures are available. The AlphaFold3 models showed high structural confidence, with an average pLDDT (predicted Local Distance Difference Test) score of 92.45 for SmeIF4A-a, and 90.28 for SmeIF4A-b. In addition, SmeIF4A-a had a pTM (predicted Template Modeling) value of 0.95 while that of SmeIF4A-b was 0.93, indicating reliable global folding. Since the binding pocket is located in a protein-RNA interface, SmeIF4A-a and SmeIF4A-b were predicted to bind in complex with polypurine (AG)_5_ RNA. Both protein-RNA complex models showed a reliable interface organization between the interacting chains with an ipTM (interface predicted Template Modeling) score of 0.94 and 0.91 for SmeIF4A-a and SmeIF4A-b, respectively. Furthermore, superposition of the models with the human eIF4A crystal structure (PDB: 5ZC9) revealed high conservation of the binding pocket. To better compare the SmeIF4A models with the human eIF4A crystal structure, rocaglamide A (RocA; first rocaglate that has been isolated^50^) was docked in the SmeIF4A-a/(AG)_5_, and SmeIF4A-b/(AG)_5_ models using GOLD, and poses were evaluated with the GOLDScore scoring function. Among the 10 generated poses, the one with the highest GOLDScore fitness value (65.46 for the SmeIF4A-a/(AG)_5_/RocA, and 60.32 for the SmeIF4A-b/(AG)_5_/RocA complex) was selected and analyzed by visual inspection. Both complexes showed good overlap with the human eIF4A/(AG)_5_/RocA crystal structure, maintaining the key interactions with both protein and RNA. The combination of high scoring values and reasonable interactions suggested quite reliable models. Docking studies of the predicted AlphaFold3 SmeIF4A-a structure^49^ in complex with polypurine (AG)_5_ RNA, and PatA indicated that PatA may also form a hydrogen bond with Glu184 similar to HseIF4A (Supplementary Fig. 1). Additionally, docking analysis of the predicted AlphaFold3 SmeIF4A structures^49^ revealed that RocA and Phe149 of SmeIF4A-a may form π-π stacking interactions similar to HseIF4A, which are crucial for the clamping mechanism of rocaglates. These interactions are missing for SmeIF4A-b because of the Leu at this position. Thus, not SmeIF4A-b but SmeIF4A-a was predicted to be rocaglate-sensitive and was therefore in the focus of further analyses.

**Fig. 2:**
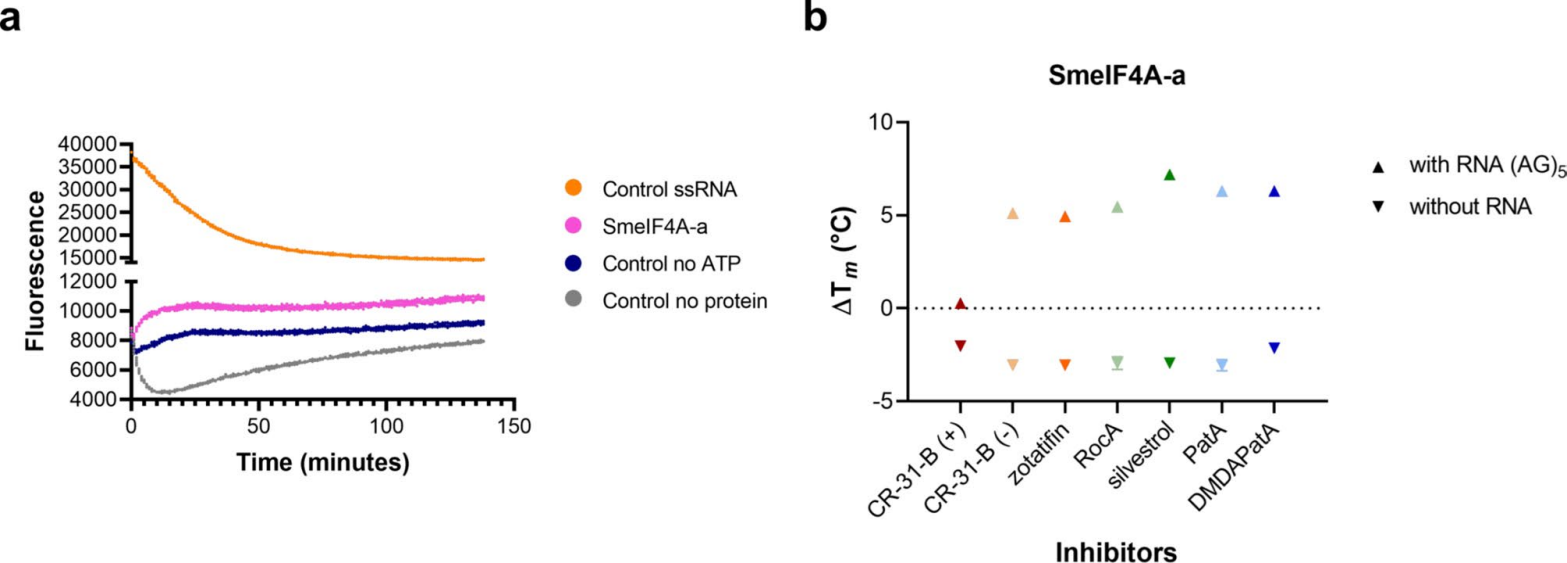
SmeIF4A-a actively unwound dsRNA and formed a complex with (AG)_5_ RNA and eIF4A inhibitors. Results of the helicase activity **(a)** and thermal shift assays (TSA) **(b)** are shown. **(a)** The helicase activity of SmeIF4A-a (magenta) was shown by an increase of fluorescence over time, and its curve was in the range between the positive and negative controls. Single-stranded RNA (orange curve) served as positive control, and two negative controls were used: (i) without protein (grey curve) and (ii) without ATP (blue curve). Unexpectedly, the latter showed a slight increase in fluorescence, which is potentially due to residual ATP amounts of *E. coli* within the purified protein fraction. **(b)** Plot of the ΔTm values after TSAs with SmeIF4A-a in complex with (AG)_5_ RNA, the non-hydrolysable ATP analog adenylyl imidodiphosphate (AMP-PNP), and different rocaglates and pateamines (upward triangles). As expected, the inactive enantiomer CR-31-B (+)^7,52^ showed no shift in thermal denaturation. All other compounds increased the thermal denaturation of SmeIF4A-a by > 5 °C. Reactions without the addition of (AG)_5_ RNA (downward triangles) demonstrated a destabilizing effect with ΔTm values < 0 °C. Data are shown as mean of a triplicate ± SEM.

To find functional evidence for the *in silico* predictions, we overexpressed SmeIF4A-a in *Escherichia coli* to perform biochemical assays. To this end, we expressed His-tagged SmeIF4A-a in *E. coli* BL21(DE3) and purified the recombinant protein by Ni-NTA affinity chromatography. The recombinant protein was then used for an established helicase assay^14,15^, which employs fluorescently labelled single-stranded RNA (Cy3-RNA) that has been annealed to its complementary RNA modified with a quencher. Finally, helicase activity is observed as an increase in fluorescence when the quencher RNA is released from Cy3-RNA. The maximum fluorescent signal was determined by Cy3-RNA without prior annealing to the quencher RNA. Additionally, two negative controls were used to define the minimum fluorescent signals of the reaction: one without protein and the other one without ATP (ATP^**−**^). ATP hydrolysis was described to be essential for the catalytic cycle of DEAD-box proteins to unwind RNA duplexes^51^. The results of the helicase assay showed an increase in fluorescence for recombinant SmeIF4A-a in between the minimum and maximum boundaries. This confirmed the expected dsRNA unwinding activity of this enzyme and its proper folding under the *in vitro* conditions used (Fig. 2). Unexpectedly, we observed a slight increase in fluorescence for the ATP^**−**^ control, which might be caused by residual ATP amounts from *E. coli* in the purified protein fraction.

Next, thermal shift assays (TSA) were performed to analyze the complex formation between SmeIF4A-a, polypurine (AG)_5_ RNA (or no RNA, as control), and rocaglates as well as pateamines. Five compounds of the rocaglate class were used, including two natural compounds, RocA and silvestrol, and three synthetically produced ones, namely zotatifin, CR-31-B (+), and CR-31-B (–). The synthesis of CR-31-B produces a racemic mixture of the two enantiomers, whereby only CR-31-B (–) displayed biological activity, and CR-31-B (+) served as control^7,52^. In addition, two different pateamines - the naturally occurring PatA and its synthetic derivative desmethyl-desamino-PatA (DMDAPatA) - were tested. As expected, the inactive enantiomer CR-31-B (+)^7,52^ caused no shift in thermal denaturation, while all other tested compounds increased the melting temperature by approximately 5 °C (Fig. 2, Supplemental Table S1). The highest shift in thermal denaturation (~ 7 °C) was observed for silvestrol.

In an analogues approach, the dsRNA unwinding activity of SmeIF4A-b was also confirmed by the helicase assay. As expected, pateamines clamp RNA onto eIF4A independent of the amino acid present at position 163 (human numbering), and caused the highest shift (~ 10 °C) of SmeIF4A-b thermal stability. However, against our expectations, all rocaglates, including the inactive enantiomer CR-31-B (+), increased the melting temperature of SmeIF4A-b by approximately 5 °C in TSAs (Supplementary Figure S2, Supplementary Table S1).

### Rocaglates and pateamines reduced the vitality of adult *S. mansoni in vitro* and impaired embryogenesis

**Fig. 3:**
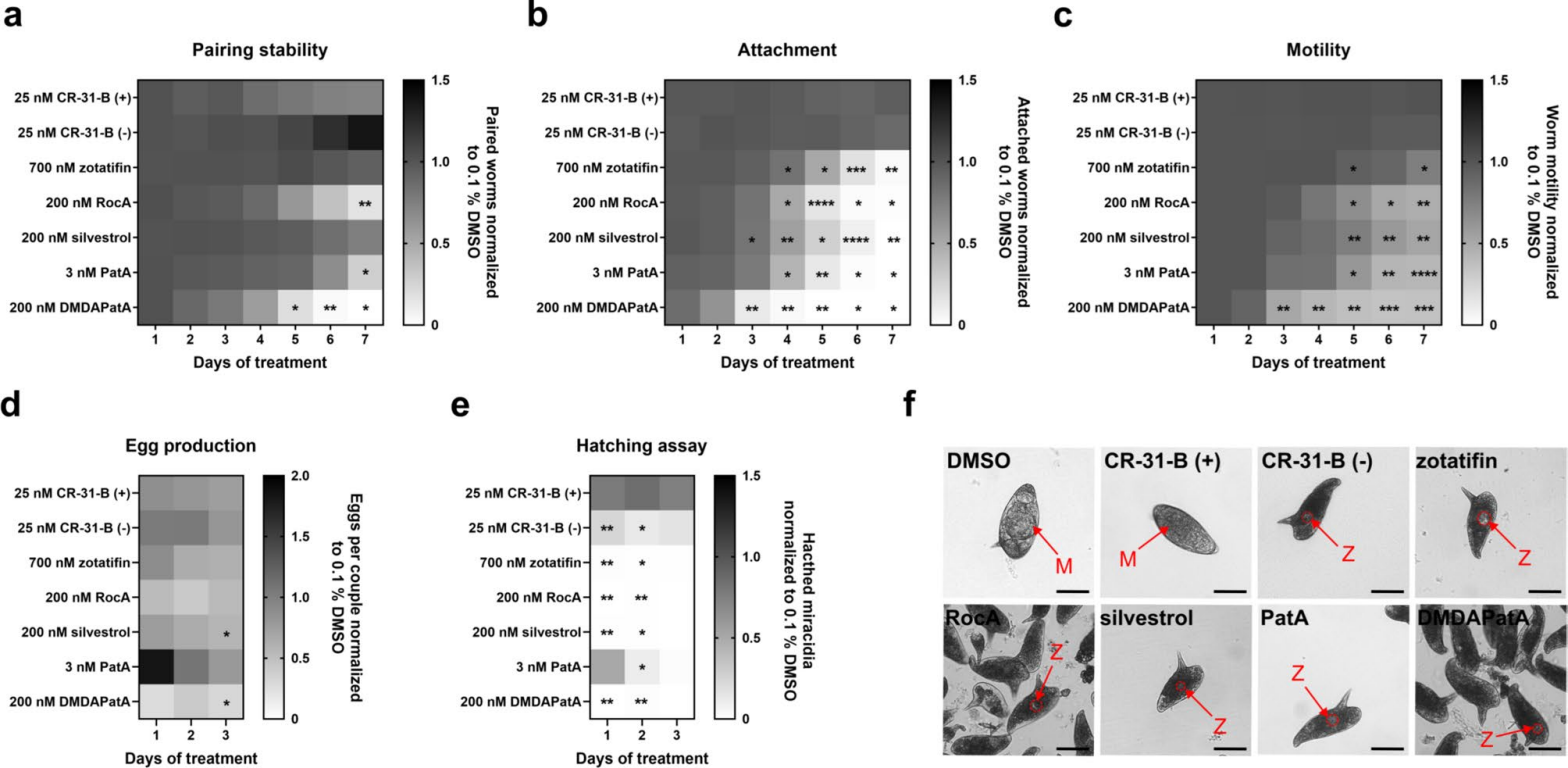
Rocaglates and pateamines reduced the vitality of adult *S. mansoni in vitro* and impaired embryogenesis. *S. mansoni* couples were treated daily with the rocaglates CR-31-B (+), CR-31-B (–), zotatifin, RocA, and silvestrol or the pateamines PatA and DMDAPatA at various concentrations over an *in vitro* culture period of 7 d. Worms incubated with 0.1 % DMSO, which is representative to all treatment concentrations, served as control. Depending on the individual compound, the following phenotypes were observed after treatment: **(a)** pairing stability was significantly reduced after RocA, PatA, and DMDAPatA treatment from day 5 on, **(b)** except for CR-31-B, all compounds significantly reduced worm attachment as well as motility **(c)** after 3–4 d with compound specific differences. **(d)** The total number of eggs decreased after 3 d of silvestrol and DMDAPatA treatment. **(e)** For the miracidia hatching assay, IVLEs of treated and control worms were first incubated for 7 d, before hatching was phototactically induced. Except for CR-31-B (+), all other compounds impaired hatching rates latest after 2 d of treatment. **(f)** Example eggs of all treatment groups after 7 d of incubation. In the DMSO (control) and CR-31-B (+) groups, miracidia-containing eggs were observed, while no further development was found in the remaining treatment groups. However, IVLEs of these treatment groups contained potential zygotes. * *p* < 0.05, ** *p* < 0.01, *** *p* < 0.001, **** *p* < 0.0001, determined by *t*-test. Scale bars: 50 μm. M = miracidium, Z = zygote.

*S. mansoni* couples were treated with either rocaglates or pateamines *in vitro*. The concentration of these compounds varied based on published concentrations active in cell models. To determine the maximum concentration to treat adult *S. mansoni in vitro*, previously published CC_50_ values of these compounds for different eukaryotic cell types (Supplementary Table S2) were used as points of reference and multiplied by the factor 10, as *S. mansoni* is a multicellular organism. In contrast to common* in vitro* compound screenings on *S. mansoni*, which apply substances in the micromolar range^53,54^ rocaglates and pateamines were tested at nanomolar concentrations. These compounds impair translation^55,56,^ which indicates a potentially delayed appearance of phenotypes depending on the turnover of affected proteins. Due to this and the low concentrations used, the treatment period was extended to 7 d. Worms were transferred daily into new 6-well plates filled with fresh medium alongside with the addition of inhibitors. Worm vitality was monitored daily by bright-field microscopy. As expected, the inactive enantiomer CR-31-B (+)^7,52^ showed no effects on worm vitality, egg production, and hatching rates of miracidia. RocA, PatA, and DMDAPatA, however, significantly reduced pairing stability between day 5–7 (Fig. 3a). Except for CR-31-B (–), all other compounds significantly reduced worm attachment to the cell-culture plate after 3–4 d, and motility after 3–5 d (Fig. 3b, c). The number of *in vitro* laid eggs (IVLEs) per worm couple was significantly lower after 3 d in the silvestrol and DMDAPatA treatment groups (Fig. 3d). The other compounds had no or only poor effects on egg production. To investigate effects of these compounds on miracidia hatching, IVLEs produced in the first 3 d of treatment were collected and separately incubated with 20 % NCS at 37 °C and 5 % CO_2_ for 7 d. After that, the medium was replaced by artificial tap water, and eggs were exposed to a light source. The overall number of eggs and hatched miracidia was counted, and the ratio of hatched miracidia calculated to investigate potential effects on embryogenesis. Except for CR-31-B (+), all used eIF4A inhibitors significantly decreased hatching rates of miracidia. PatA had a delayed effect and reduced miracidia-hatching rates from eggs laid on day 2, while the other active compounds showed negative effects on hatching already for eggs laid on day 1 (Fig. 3e). Despite hatched miracidia, the remaining IVLEs were microscopically analyzed to determine their developmental stages^57^. IVLEs of all treatment groups contained potential zygotes, but further developed eggs with a visible larva (miracidium) were only found in the DMSO control and the CR-31-B (+) group (Fig. 3f). CR-31-B (–) impaired embryogenesis, although no effects on adult worms were observed within 7 d of treatment.

Except for silvestrol^14^, none of the rocaglates and pateamines used in this study have been tested against a metazoan parasite before. Therefore, we additionally investigated uptake routes of these compounds in adult *S. mansoni.* For this, we applied atmospheric-pressure scanning microprobe matrix-assisted laser desorption/ionization mass spectrometry imaging (AP-SMALDI MSI), as previously described^58^. Zotatifin was the only eIF4A inhibitor that was ionizable and detectable using this method. Zotatifin appeared to be distributed along the esophagus and the intestine of male and female *S. mansoni*, but it was not detected in the tegument. After 1 d of treatment, zotatifin was distributed throughout the entire parasite (Supplementary Figure S3), and remained distributed also at later time points (3, 5, 7 d; data not shown).

### Specific rocaglates and pateamines impaired spermatogenesis, oogenesis, and vitellogenesis

**Fig. 4:**
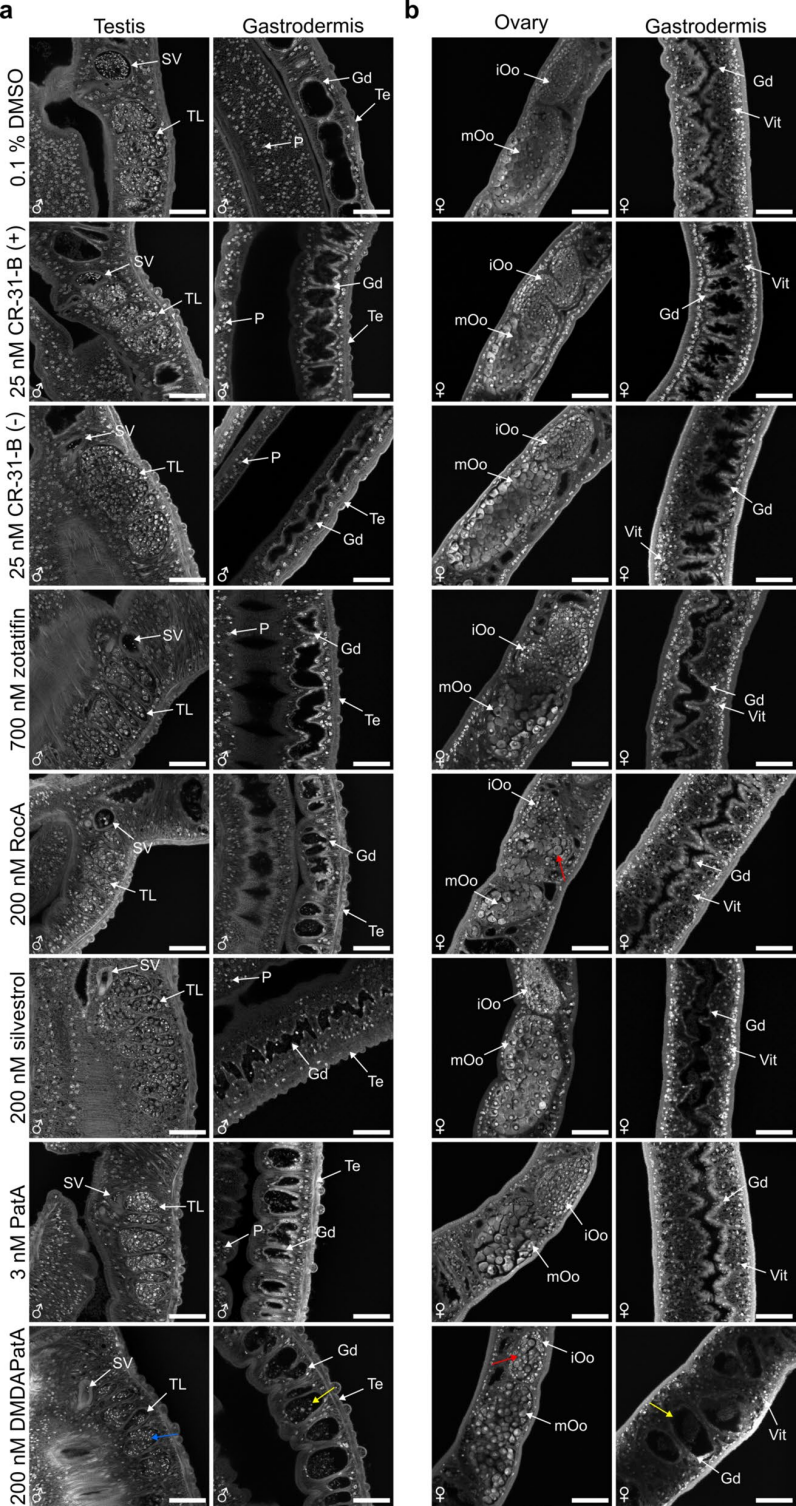
Morphological analyses of eIF4A inhibitor-treated *S. mansoni* males and females showed various effects. CLSM analysis of the reproductive organs (testis and ovary) as well as the gastrodermis of males **(a)** and females **(b)** after separation of the couples. Zotatifin-, RocA-, and silvestrol-treated males showed less mature sperm in the testicular lobes. The testis of DMDAPatA-treated males was mainly filled with mature sperm (blue arrow), and the intestine was enlarged. Furthermore, aggregates of the gastrodermis were found in the gut lumen (yellow arrows). Less oogonia were observed in the ovaries of females treated with RocA and DMDAPatA (red arrows). Scale bars: 50 μm. Gd = Gastrodermis, iOo = oogonia, mOo = mature oocyte, SV = seminal vesicle, Te = tegument, TL = testicular lobes, Vit = vitellarium. Representative images of three independent experiments (*n* = 3) with three worms per experiment are shown.

After 7 d of treatment, couples were separated, and males and females stained by carmine-red to be morphologically analyzed by confocal laser scanning microscopy (CLSM). In this context, especially the testis and ovary as well as the intestine of males and females were examined. In untreated males, the testicular lobes are filled with stem-cell like spermatogonia, primary and secondary spermatocytes, spermatids, and mature sperm. The latter occur at a higher density at the ventral site of the lobes, where sperm enters the *vas deferens* and then accumulates in the seminal vesicle^59^. In males treated with zotatifin, silvestrol, and RocA, the number of mature sperm seemed to be reduced in the testicular lobes as well as in the seminal vesicle. Instead, the testes were mainly filled with spermatogonia and spermatocytes. In contrast, less spermatogonia and spermatocytes were observed in DMDAPatA-treated males, in which the testicular lobes were predominantly packed with spermatids and mature sperm. A similar impression was obtained in males treated with PatA, albeit to a lesser extent (Fig. 4a).

The ovary of females is divided into an immature and mature part. The immature part is located anterior and is filled with stem-cell like oogonia, while the larger part at the posterior site is filled with mature, differentiated oocytes. Cells in transition are found between both parts^60,61.^ The number of oogonia was reduced in females treated with RocA and even more after DMDAPatA treatment. In these females, mature oocytes were not only present in the posterior part of the ovary but were also in its anterior part. Additionally, in DMDAPatA-treated males and females the gut lumen was clearly enlarged and aggregates of gastrodermis tissue were observed in the gut lumen (Fig. 4).

**Fig. 5:**
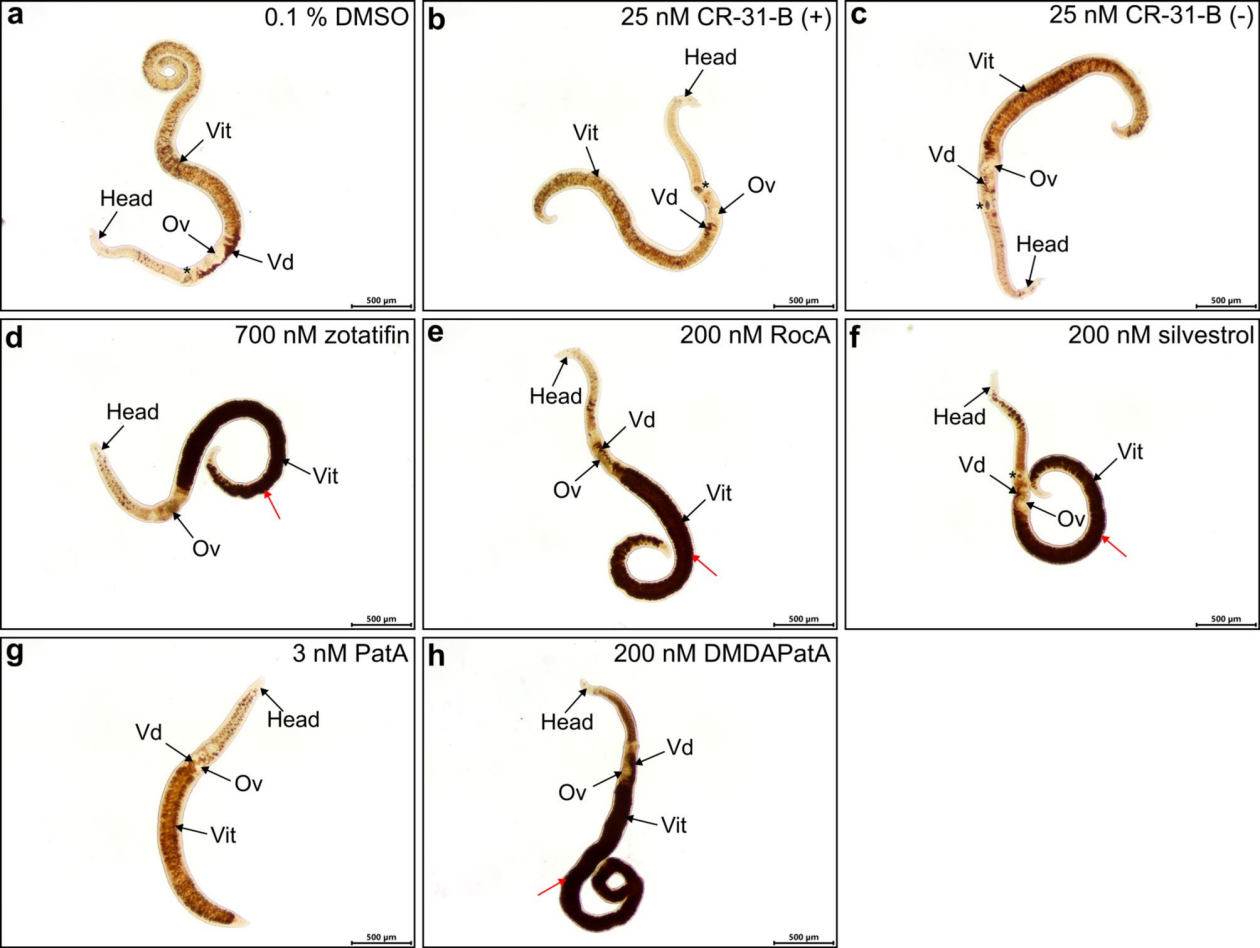
Treatment with zotatifin, RocA, silvestrol, and DMDPatA affected vitellogenesis of *S. mansoni* females. After 7 d of treating adult *S. mansoni in vitro*, females were separated from males, and lipid droplets were stained by Oil-red O. Both CR-31-B enantiomers as well as PatA-treated females showed staining intensities comparable to control females (DMSO-treated) **(a-c; g)**. The vitellarium of females treated with zotatifin **(d)**, RocA **(e)**, silvestrol **(f)**, and DMDAPatA **(h)** appeared to be darker (red arrows). Ov = ovary, Vd = vitelloduct, Vit = vitellarium. The asterisk (*) marks an egg in the ootype. Representative images of three independent experiments (*n* = 3) with two worms per experiment are shown.

The vitellarium of *S. mansoni* females is densely filled with vitellocytes of the stages S1-S4. While S1 vitellocytes have stem cell characteristics, S2-S3 vitellocytes are differentiating, and S4 vitellocytes are terminally differentiated^62,63.^ S3/S4 vitellocytes contain lipid droplets, which enables the monitoring of their differentiation status by staining with Oil-Red O^64,65^. The vitellarium of females treated with both CR-31-B enantiomers and PatA showed similar staining intensities compared to the DMSO control. In these worms, highest staining intensities were observed in the vitelloduct, which transports S4 vitellocytes from the vitellarium to the ootype for egg formation. In females treated with zotatifin, RocA, silvestrol, and DMDAPatA, however, the vitellarium appeared to be darker than in control females. Furthermore, the vitellarium was homogenously stained preventing the possibility to distinguish the vitelloduct from the surrounding tissue. This indicated a shift towards more S3/S4 vitellocytes after treatment with zotatifin, RocA, silvestrol, or DMDAPatA (Fig. 5).

### Rocaglates and pateamines decreased stem-cell proliferation in adult *S. mansoni* and induced apoptosis

**Fig. 6:**
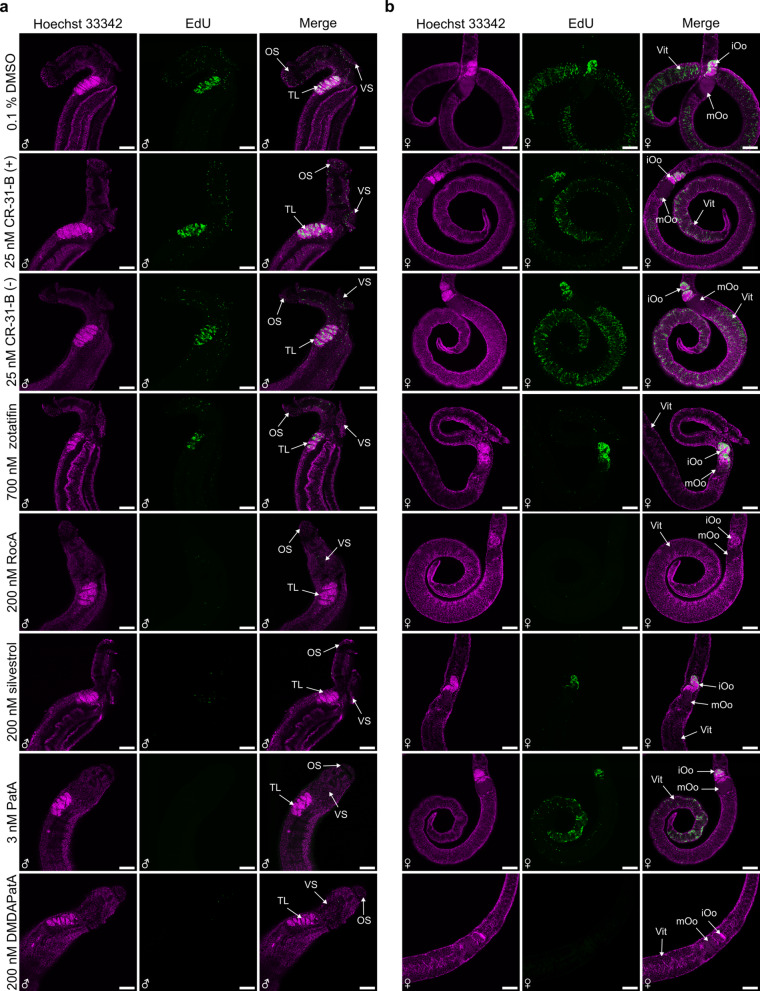
Rocaglates and pateamines reduced stem-cell proliferation in male and female *S. manoni*. Both enantiomers of CR-31-B had no effects on stem-cell proliferation. Males **(a)** and females **(b)** of these groups showed similar amounts of EdU signals (green) compared to the control (DMSO). All remaining treatment groups (zotatifin, RocA, silvestrol, PatA, and DMDAPatA) showed less EdU^+^ cells in males and females with a stronger effect on males. Total DNA was stained by Hoechst 33342 (magenta). Scale bars: 100 μm. iOo = oogonia, mOo = mature oocyte, OS = oral sucker, TL = testicular lobes, Vit = vitellarium, VS = ventral sucker. Representative images of three independent experiments (*n* = 3) with three worms per experiment are shown.

The life cycle of *S. mansoni* as well as their longevity in the final host depend on the activity of GSCs and SSCs^37–40.^ Therefore, examining stem-cell proliferation after inhibitor treatment is a valuable approach to assess the potential of active substances against *S. mansoni in vitro*. After 7 d of inhibitor treatment, worms were incubated for 24 h with 5-ethynyl-2’-deoxyuridine (EdU), which is a thymidine analog and incorporated during the S-phase into synthesized DNA^66^. The EdU assay and subsequent CLSM analyses exhibited a reduction of stem-cell proliferation of SSCs and GSCs in all groups, except for both CR-31-B enantiomers. The number of EdU^+^ cells in testis and ovary was significantly reduced after treatment with zotatifin, RocA, silvestrol, PatA, and DMDAPatA (Fig. 6; Table 1). A stronger reduction of EdU^+^ cells was observed in testes than in ovaries in these treatment groups. In females treated with zotatifin, silvestrol, and PatA, few EdU^+^ cells were observed, while almost no EdU^+^ cells were detected in RocA- and DMDAPatA-treated females (Fig. 6; Table 1).

**Table 1:** EdU^+^ assays revealed stem-cell effects in adult *S. mansoni*. The number of EdU^+^ cells per mm^2^ (mean ± SD) in testis or ovary, respectively, was determined and normalized to the DMSO control. Except for the CR-31-B treatment, all other compounds significantly reduced the number of EdU^+^ cells in the testis and ovary. * *p* < 0.05, *** *p* < 0.001, **** *p* < 0.0001, determined by *t*-test.

Compounds	Testis			Ovary		
	EdU^+^ cells/mm^2^	Normalized to 0.1 % DMSO	Statistically significant?	EdU^+^ cells/mm^2^	Normalized to 0.1 % DMSO	Statistically significant?
CR-31-B (+)	8.99 ± 1.73	0.95	No	9.37 ± 1.58	1.08	No
CR-31-B (–)	8.86 ± 0.96	0.94	No	8.08 ± 2.10	0.93	No
Zotatifin	4.74 ± 2.41	0.52	Yes, ***	7.40 ± 2.59	0.72	Yes, *
RocA	0.16 ± 0.17	0.02	Yes, ****	0.48 ± 0.43	0.06	Yes, ****
Silvestrol	0.37 ± 0.60	0.04	Yes, ****	6.87 ± 3.10	0.67	Yes, *
PatA	0.02 ± 0.05	0.00	Yes, ****	4.08 ± 1.51	0.47	Yes, ****
DMDAPatA	0.00 ± 0.00	0.00	Yes, ****	0.90 ± 1.44	0.10	Yes, ****

In addition to the anti-proliferative effects of rocaglates and pateamines described in cell lines, induction of apoptosis has also been reported^16,67–69.^ We investigated the induction of apoptosis by a terminal deoxynucleotidyl transferase dUTP nick-end labeling (TUNEL) assay on adult *S. mansoni* after 7 d of treatment. Both CR-31-B enantiomers were excluded, as CR-31-B (–) did not provoke any effects on adult *S. mansoni*. In both males and females, more apoptotic cells were observed after rocaglate and pateamine treatment than in the control group (DMSO). However, no difference in apoptotic signals was observed between these two compound classes (Supplementary Figure S4).

### Rocaglates caused reversible effects on adult *S. mansoni*, while pateamines irreversibly impaired worm vitality and stem-cell proliferation *in vitro*

**Fig. 7:**
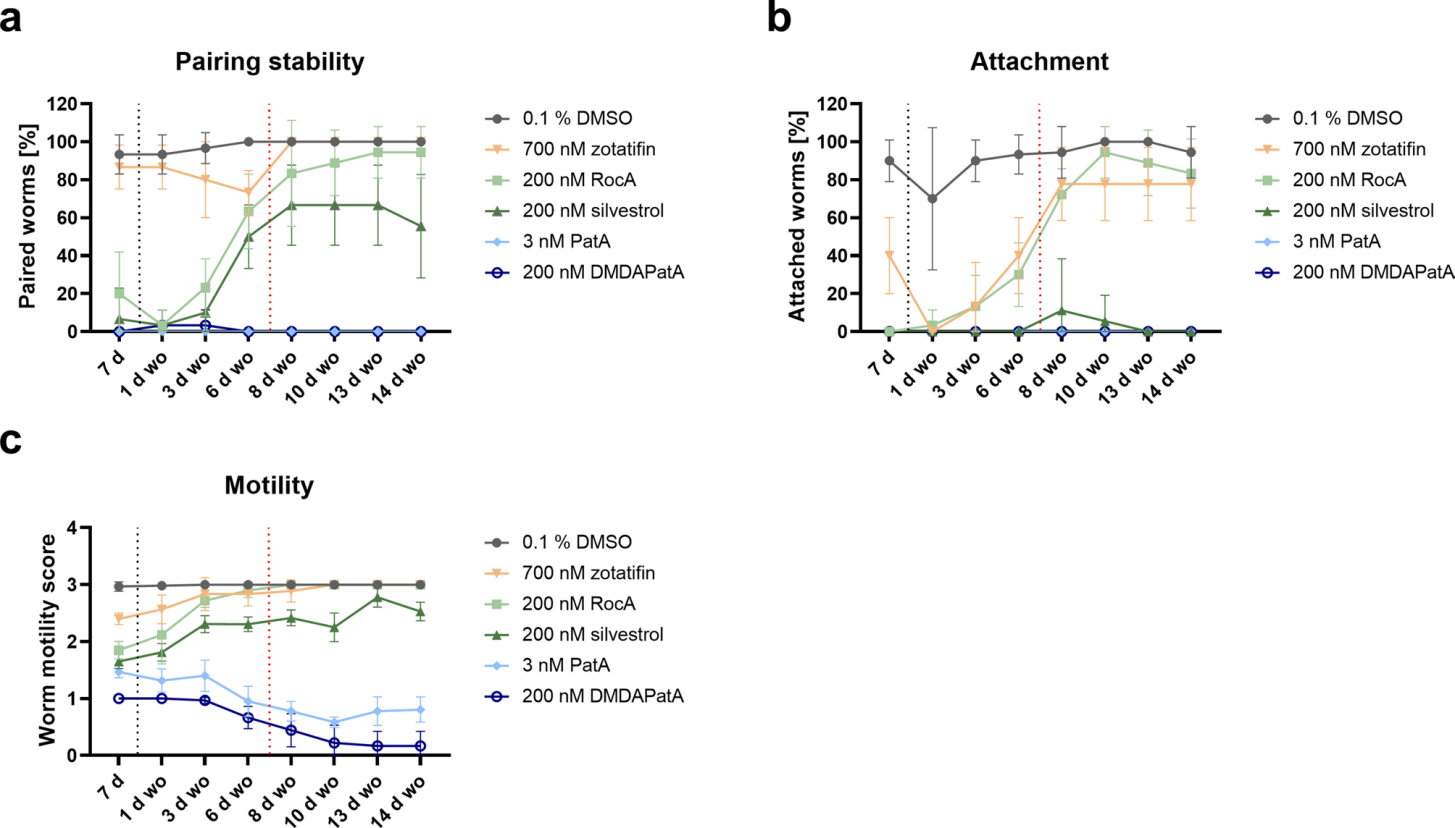
*S. mansoni* couples recovered from rocaglate but not from pateamine treatment. Five *S. mansoni* couples were treated daily with the rocaglates zotatifin, RocA, and silvestrol or the pateamines PatA and DMDAPatA for 7 d *in vitro*. Then, compounds were washed out by incubating the worms in blank medium (black dashed line) for up to 14 d. After 7 d of washout (wo), two couples of each approach were incubated with EdU for 24 h, and finally fixed for the EdU stem-cell proliferation assay (red dashed line). The remaining three couples were incubated until day 14. The medium was replaced every 2–3 d. The following phenotypes were observed: **(a)** the number of paired worms increased over time after rocaglate wo, while worms remained separated after pateamine wo; **(b)** The attachment of *S. mansoni* to the surface of the petri dish increased again after zotatifin and RocA wo; **(c)** worm motility increased after rocaglate wo, while it further declined for pateamine-treated worms. Most of the DMDAPatA-treated worms were classified as dead after 14 d of wo.

Although both rocaglates and pateamines have been shown to target eIF4A in the same pocket^11^ exhibiting anti-proliferative and pro-apoptotic effects in cell lines *in vitro*, PatA was reported to irreversibly inhibit the proliferation of various cell lines, while the inhibitory effects of the rocaglate silvestrol were reversible^56,70,71.^ To address this point, we investigated the reversibility of rocaglate or pateamine treatment on the vitality and stem-cell proliferation. To this end, adult *S. mansoni* were treated daily with rocaglates and pateamines for 7 d *in vitro* and subsequently incubated in blank medium for up to 14 d, as wash-out (wo) simulation. Except for zotatifin (*n* = 3), wo experiments of each compound were done with six biological replicates (*n* = 6). During wo, worms were analyzed for different vitality parameters as mentioned before. Wo of zotatifin, RocA, and silvestrol increased the number of paired worms and worm motility. While wo after zotatifin and RocA treatment restored worm attachment, worms remained unattached after silvestrol wo (Fig. 7). In this experiment, silvestrol also affected the pairing status after 7 d of treatment, which is in contrast to previous observations (Fig. 3). The reason for this might be a different potency of the silvestrol batch used for the wo experiment. Unlike rocaglates, pateamine wo failed to rescue the vitality of adult *S. mansoni*. In fact, worm motility continued to decline after pateamine wo, and most worms of the DMDAPatA group were classified as dead after 14 d of wo (Fig. 7). These results indicate that adult *S. mansoni* recovered from rocaglate treatment, while pateamines irreversibly impaired parasite vitality *in vitro* .

**Fig. 8:**
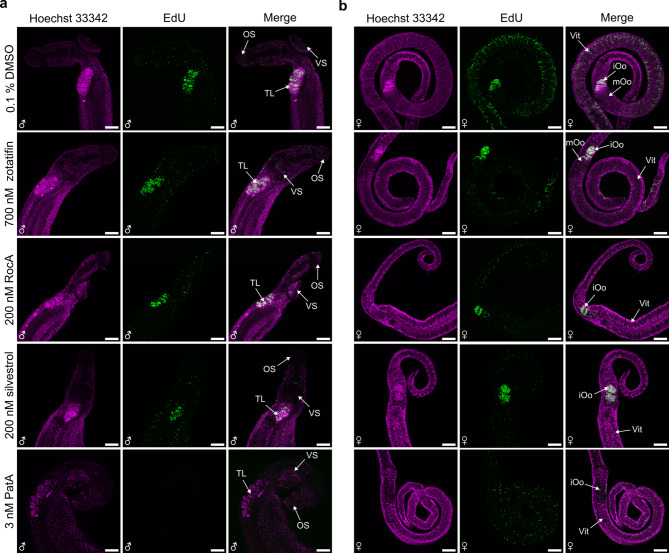
Washout of rocaglate treatment rescued stem-cell proliferation in male and female *S. mansoni*. Couples were treated daily with the rocaglates zotatifin, RocA, and silvestrol or the pateamines PatA and DMDAPatA for 7 d *in vitro*. Then, worms were cultured for 14 d without the addition of compounds, and the medium was replaced every 2–3 d. After 14 d of wo, three couples of each approach were incubated with EdU for 24 h. An EdU assay was performed and the worms analyzed by CLSM. Total DNA was stained by Hoechst 33342 (magenta). EdU signals (green) were observed in males and females of the rocaglate wo groups, while no EdU^+^ cells were detected in PatA-treated males, and to a low extent in females. Scale bars: 100 μm. iOo = oogonia, mOo = mature oocyte, OS = oral sucker, TL = testicular lobes, Vit = vitellarium, VS = ventral sucker. Representative images of three independent experiments (*n* = 3) with three worms per experiment are shown.

Next, we performed EdU assays after 7 and 14 d of wo to analyze whether stem-cell proliferation was restored after compound withdrawal. After 7 d of wo, two couples of each approach were incubated with EdU and analyzed by CLSM as described above, while the remaining three couples were used for the EdU assay after 14 d of wo. In total, EdU assays were performed for three biological replicates (*n* = 3) of each compound used. After 7 d of wo, proliferating EdU^+^ cells were observed in males and females of the rocaglate groups. While a high density of EdU^+^ cells was present in the testis and ovary after zotatifin wo, lower densities were detected in the testis and ovary of the RocA and silvestrol groups (Supplementary Figure S5). This was consistent with the vitality parameters, which showed a greater reduction of parasite vitality after treatment with RocA or silvestrol compared to zotatifin (Fig. 7), as well as with the previously observed stronger effects on stem-cell proliferation (Fig. 6). Therefore, recovery after incubations with RocA or silvestrol might take longer than after treatment with zotatifin. Some EdU^+^ cells were observed in females of the PatA wo group, but they were absent in males. Since most DMDAPatA-treated worms were dead, no EdU assays could be performed for this experimental group. After 14 d wo, a higher density of EdU^+^ cells in the testis and ovary of the RocA and silvestrol groups was detected indicating increased proliferation at this later time point. In contrast, in the PatA group, no increase in EdU^+^ signals was observed at 14 d wo compared to 7 d wo (Fig. 8).

## Discussion

For *Aglaia* plants, a protective function or feeding protection by endogenous rocaglates against pathogens or pests was discussed. This was supported by the findings that *Aglaia* plants harbor an eIF4A variant with F163L, which leads to rocaglate non-sensitivity and thus prevents self-poisoning^10^. A fungus of the genus *Ophiocordyceps* was found to parasitize *Aglaia* plants and carries the rocaglate-insensitive binding motif TPGFQI, which is potentially the result of co-evolution^72^. Unfortunately, no functional data on PatA and the eIF4A sequence from *M. hentscheli* have been generated yet. This functional mimicry by an unrelated organism indicates an advantage of these organisms to synthesize molecules that target eIF4A of potential pests. Over the past two decades, the anti-viral and anti-parasitic potential of rocaglates has been further investigated. This included the natural rocaglate silvestrol, which reduced the vitality of adult *S. mansoni in vitro*^14^.

The TSA results of SmeIF4A-a, (AG)_5_ RNA, and rocaglates as inhibitors confirmed the prediction of rocaglate-sensitivity. Silvestrol has an additional dioxanyloxy-moiety, which interacts with an Arg-rich pocket of the target^14^. Two of these Arg residues are present in SmeIF4A-a and might explain that silvestrol caused the highest thermal stabilization of this protein from the rocaglate compound class. Furthermore, (AG)_5_ RNA was clamped on SmeIF4A-a by pateamines, which are a promising compound class because they do not depend on Phe163. This reduces the risk of escape mutations and broadens the range of applications, as demonstrated by the effective treatment of the rocaglate-resistant pathogen *L. major* with PatA^15^.

The binding of rocaglates to SmeIF4A-b, which harbors the TPLFQI motif, was rather unexpected (Supplementary Figure S2). Similar results were obtained for a mutated eIF4A variant (H161L) from the mosquito *Aedes aegypti* (binding motif: TPLFQV), which showed increased thermal stability after the addition of rocaglates in TSA. To explain this, the influence of a seventh amino acid in the binding pocket was discussed. Mutated human eIF4A (F163L) was found to be insensitive to rocaglates, and this variant has an Asn167 that seemed to close the rocaglate binding pocket. In case of *Ae. aegypti*, Ser165 is present at this position, which might keep the binding pocket more open^73^. SmeIF4A-b has also a serine (Ser150) within the binding pocket, similar to *Ae. aegypti*. Docking analysis showed a broader rocaglate binding pocket compared to SmeIF4A-a and human eIF4A with an asparagine (Supplementary Figure S6). This might explain that SmeIF4A-b can still interact with rocaglates, but the influence of Ser150 on the binding pocket of SmeIF4A-b needs to be examined in more detail in future studies.

The earliest effects of zotatifin on adult *S. mansoni* were observed after 4 d of *in vitro* culture. AP-SMALDI MSI measurements indicated that this may not be due to a delayed or uneven distribution of the compound within the parasite. We rather assume biological reasons related to (target) protein half-life and eIF4A function, which implies translation initiation. This might be altered upon eIF4A inhibition with delayed physiological and morphological outcomes.

Although the effects of rocaglates and pateamines are difficult to compare because they were applied at different concentrations, DMDAPatA showed the earliest onset of inhibitor treatment (reduced motility on day 3), while the rocaglates RocA and silvestrol, used at the same concentration (200 nM), reduced the motility after 5 d of treatment. Additionally, different morphological changes were observed after treatment with rocaglates or pateamines. While spermatogonia and spermatocytes but no differentiated sperm were observed in the testis of rocaglate-treated worms, mainly mature sperm occurred in the testis after DMDAPatA treatment. This suggests that the self-renewal of spermatogonia was blocked, but not differentiation processes that ultimately lead to mature sperm. This was confirmed by EdU assays showing reduced stem-cell proliferation of GSCs and SSCs for both compound classes. Overall, the anti-proliferative properties of these compounds previously demonstrated in cancer cell lines^16,56^ were also found in *S. mansoni*.

Inhibition of eIF4A by rocaglates has been linked to cell-cycle disruption. While treatment of LNCaP cells with silvestrol arrested the cell cycle at the G2/M transition^74^, a blockage of G1/S phase transition was described in MDA-MB-231 cells^75^. These observations are consistent with our findings showing a reduction of EdU^+^ cells after rocaglate or pateamine treatment, as EdU is incorporated during the S-phase^66^. Furthermore, the increased lipid-staining intensities after treatment with zotatifin, RocA, silvestrol or DMDAPatA were in line with reduced stem-cell proliferation and indicated a shift towards more S3/S4 instead of S1/S2 vitellocytes. In addition, the reduced number of miracidia after rocaglate or pateamine treatment points to impaired embryogenesis, a process that involves many cell-proliferation steps^57^. Overall, these inhibitor-induced phenotypes correspond to our findings after knockdown of Sm*eif4a-a* by RNA interference^45^, and together with the TSA results, our results strongly suggest that both compound classes target SmeIF4A-a. In humans, eIF4A activity was found to affect about 300 mRNAs, which are characterized by long, structured 5’-UTRs. These mRNAs represent genes involved in cell-cycle regulation like cyclins and cyclin-dependent kinases^7,75^. In *S. mansoni*, the identity of SmeIF4A-dependent mRNAs remains unknown at this stage of the analysis, but our results on reduced cell proliferation point towards similar cell-cycle regulators. The broader diversity of phenotypes observed after DMDAPatA compared to rocaglate treatment might be explained by the earlier onset of eIF4A inhibition following treatment. In contrast to rocaglates, pateamines are not dependent on a polypurine stretch in their mRNA target for efficient RNA clamping^15^. This might enlarge the pool of mRNA substrates that can be clamped by pateamines, and it may explain the earlier anti-schistosomal activity following DMDAPatA treatment.

Impairment of the gastrodermis tissue accompanied by aggregate formation observed after DMDAPatA treatment phenocopied effects seen after the treatment of adult *S. mansoni* with arylmethylamino steroids. These compounds potentially chelate metals and may be involved in haem bioactivation^76^. Among the arylmethylamino steroids, compound sc1o was shown to affect cell viability by cell-cycle arrest at G1, which provides a first phenotypic link between this compound class and pateamines^77^.

Compared to other compounds tested in our study, the synthetic rocaglate CR-31-B (–) failed to induce multifaceted phenotypes in adult *S. mansoni*, but it significantly decreased the number of hatched miracidia. Previous bulk RNA-seq data of all *S. mansoni* life stages^78^ revealed approximately 5-fold higher transcript levels of Sm*eif4a-a* in the egg stage compared to adult worms (Supplementary Figure S7). This elevated requirement of SmeIF4A-a in the egg stage might explain the impaired embryogenesis after CR-31-B (–) treatment, while the concentration might have been too low to show any effects on adult worms *in vitro*.

The reversibility of rocaglate treatment identified by wo experiments suggests that although apoptotic cells were detected, other cells were potentially only arrested (or not yet dead) and able to re-enter the cell cycle. Against our expectations, there was no difference in apoptotic signals between the pateamine and rocaglate treatment groups even though treatment with pateamines was irreversible. We analyzed apoptotic cells after 7 d of treatment, which might have been too late to detect greater DNA fragmentation in DMDAPatA-treated worms, where inhibitor treatment effects started earliest. In future studies, an earlier time point e.g., 4 d, should be considered to analyze potential differences between rocaglates and pateamines that may uncover reversible or irreversible effects of these compounds. To our knowledge, the exact mechanism leading to irreversible effects of pateamines has not yet been clarified in any study.

The irreversible mechanism of pateamines appears to be beneficial in the treatment of adult *S. mansoni in vitro*, as most worms died. But these effects may be unfavorable in terms of potential application. For  *in vivo* studies, the reversible effects of rocaglates might be more suitable to reduce toxic side effects on human eIF4A. The fact that rocaglates and pateamines also target human eIF4A^10,11,^ however, represents a limitation of these compounds as anti-schistosomal candidates. Zotatifin showed activity against adult *S. mansoni in vitro*, but the natural rocaglates silvestrol and RocA seemed to impair worm vitality more potently. This could be caused by a different pool of mRNAs clamped by these compounds. The dioxane moiety of silvestrol and its interaction with the Arg-rich pocket enables the clamping of mRNAs without a polypurine stretch^79^. However, silvestrol was shown to be a substrate of the p-glycoprotein efflux pump, resulting in poor bioavailability. Therefore, local administration of silvestrol was proposed as anti-viral approach^80^. This is not applicable for the treatment of *S. mansoni*, which reside in the mesenteric veins of the intestine. Thus, anti-schistosomal candidates should be formulated for oral administration^81^. Overall, the pharmacological properties of rocaglates and pateamines impede their suitability for further development as anti-schistosomal candidates. However, the target SmeIF4A-a might remain an interesting option, as its inhibition blocks life-cycle progression, egg production, and reduces parasite vitality.

In summary, our study demonstrated that several rocaglates (silvestrol, RocA, and zotatifin) as well as the pateamines PatA and DMDAPatA target SmeIF4A-a. Treatment reduced parasite vitality and stem-cell activity in the nanomolar range *in vitro*. Except for silvestrol^14^, this was the first time that these naturally derived eIF4A inhibitors were tested and analyzed in more detail for their effects against a metazoan parasite.

## Methods

### Docking analyses

AlphaFold3 was used to predict the structure of both SmeIF4A isoforms (SmeIF4A-a, Smp_097660; SmeIF4A-b, Smp_166400)^49^. The full-length amino acid sequences of SmeIF4A-a and SmeIF4A-b were used as input together with the polypurine RNA (AG)_5_, Mg^2+^, and ATP. Four models were generated for each protein isoform, and the model with the highest average pLDDT score was selected for docking analyses (92.45 for SmeIF4A-a, and 90.28 for SmeIF4A-b). SmeIF4A-a showed a pTM score of 0.95 and an ipTM score of 0.94, while that of SmeIF4A-b were 0.93 and 0.91, respectively.

Docking analyses were performed using the program GOLD (Genetic Optimization for Ligand Docking)^82^. The protein structures were prepared using Hermes implemented in the GOLD Suite. The binding site was defined as residues located within 10 Å of the predicted active-site region, and contained the rocaglate binding motif TPFFQI (Thr144, Pro145, Phe149, Phe178, Gln181, Ile185) in case of SmeIF4A-a or TPLFQI (Thr141, Pro142, Leu146, Phe175, Gln178, Ile182) for SmeIF4A-b, respectively, the Arg-rich pocket (Arg97, Val268, Arg297 for SmeIF4A-a; Arg94, Arg265, Arg294 for SmeIF4A-b), as well as (AG)_5_ RNA in the RNA binding pocket. The 3D conformer of RocA was retrieved from PubChem (CID 331783) in SDF format. The ligand was prepared prior to docking by adding hydrogen atoms (pH 7.4) and performing energy minimization in MOE (219.0102). Molecular docking was subsequently carried out using GOLD with the empirical GOLDScore scoring function. During a single docking run, 10 poses were generated, and the one with the highest GOLDScore fitness value (65.46 for the SmeIF4A-a/(AG)_5_/RocA, and 60.32 for the SmeIF4A-b/(AG)_5_/RocA complex) was further analyzed in the PyMOL Molecular Graphics System (v 3.1.4.1; Schrödinger, LLC), which was used for both, the analysis of the predicted AlphaFold3 structures, and for image generation.

### RNA isolation, cDNA synthesis, and cloning of SmeIF4A

RNA from *S. mansoni* couples was extracted using the Monarch^®^ Total RNA Mini-prep Kit (NEB, UK) according to the manufacturer’s instructions. The quality and quantity of isolated RNA were analyzed by electropherograms (Bioanalyzer 2100; Agilent Technologies, USA), and reverse transcribed into cDNA with the QuantiTect^®^ Reverse Transcription Kit (Qiagen, Germany). The coding sequences (CDS) of Sm*eif4a-a* and Sm*eif4a-b*, respectively, were amplified with primers designed to create overhangs for Gibson assembly^83^ (primers: SmeIF4A-a_fw: 5’-gtg ccg cgc ggc agc CAT ATG AGT AAT TCT GTA GAA GAT TCT GA-3’, SmeIF4A-a_rev: 5’-gtg gtg gtg gtg CTC GAG TTA CAG AAA ATC AAC AAT GTC ATC AGG-3’, SmeIF4A-b_fw: 5’- gtg ccg cgc ggc agc CAT ATG GAG TCG GAC AAC AAC ATA AAC-3’, SmeIF4A-b_rev: 5’-gtg gtg gtg gtg CTC GAG TTA GAA TAA ATT AGC TAC ATC CAT TGG TA; lower case letters represent overlaps with the vector). The 50 µl PCR reaction consisted of: 10 µl Q5 reaction buffer (NEB, UK), 1 µl dNTPs (10 mM; Solis BioDyne, Estonia), 1.5 µl ethylene glycol (Merck, Germany), 0.5 µl Q5 High-Fidelity Polymerase (NEB, UK), 10 ng template DNA, and 2.5 µl of gene-specific primers (10 µM each). Samples were amplified as two-step PCR with the following program: initial denaturation at 98 °C for 3 min, 10 cycles of denaturation at 95 °C for 30 s, annealing at 58 °C for 30 s, elongation at 72 °C for 1 min, followed by 25 cycles of denaturation, annealing, and elongation whereas the annealing temperature was increased to 68 °C. PCR success was confirmed by agarose gel electrophoresis, and the resulting amplicon was purified using the Monarch PCR & DNA Cleanup Kit (NEB, UK) according to the manufacturer’s instructions.

For cloning, the recombinant plasmid pET28a(+)_HseIF4A1_(19–406)^84^ was digested by the restriction enzymes *Nde*I and *Xho*I (NEB, UK) to replace human *eif4a* by Sm*eif4a-a*, and Sm*eif4a-b*, respectively. A 1:2 ratio of linearized vector and insert were incubated with the HiFi DNA Assembly Master Mix (NEB, UK) at 50 °C for 40 min. Subsequently, recombinant plasmids were transformed into *E. coli* 10-beta (NEB, UK) by heat-shock at 42 °C for 80 s. Cells were grown at 37 °C for 1.5 h and spread on lysogeny broth (LB) agar plates containing 50 µg/ml kanamycin as selection marker. Plates were incubated at 37 °C overnight. Single clones were picked to inoculate LB medium supplemented with 50 µg/ml kanamycin. Plasmids were isolated by alkaline lysis^85^, purified by the Monarch PCR & DNA Cleanup Kit (NEB, UK), and their sequence confirmed by sanger sequencing (Microsynth SeqLab, Germany). After sequence confirmation, recombinant plasmids were transformed into competent *E. coli* BL21(DE3) as protein expression strain. Bacterial clones were stored in 25 % glycerol at −80 °C.

### Recombinant protein expression and purification

Glycerol stocks of SmeIF4A-a_pET28a(+) and SmeIF4A-b_pET28a(+) were used to inoculate pre-cultures, which were incubated at 37 °C and 140 rpm overnight. Eight 1 L Erlenmeyer flasks filled with 200 ml LB medium, and 50 µg/ml kanamycin were inoculated to an OD_600_ = 0.1 using the pre-culture. Bacterial cultures were grown at 37 °C until they reached the mid-log phase (OD_600_ = 0.6–0.8). After the addition of 0.5 mM IPTG to induce protein expression, cells were grown at room temperature (RT; 22–23 °C) overnight. Cells were harvested by several rounds of centrifugation at 4,500 g and 4 °C for 10 min each. The supernatant was discarded, cell pellets frozen in liquid nitrogen and stored at −80 °C.

Cell pellets were resuspended in lysis buffer consisting of 20 mM HEPES pH 7.5, 300 mM KCl, 20 mM imidazole, 10 mM β-mercaptoethanol, and 10 % (v/v) glycerol. Two tablets of cOmplete™ Mini Protease Inhibitor Cocktail, EDTA-free (Roche, Germany) as well as 0.125 mg/ml lysozyme and 0.5 µl Benzonase^®^ Nuclease (Merck, Germany) were added to the solution until the tablets were completely dissolved. Cells were lysed by sonication on ice (10 × 1 min with 2 min breaks, 50 % power; Sonifier 250, Branson) and centrifuged at 20,000 rpm at 4 °C for 60 min (Avanti JXN-26, Beckman Coulter). The supernatant was passed through a 0.45 μm filter (Filtropur S, Sarstedt). Protein purification was performed using UNICORN™ (v 7.3) on an ÄKTA go™ chromatography system (Cytiva, USA). Samples were loaded onto a 1 ml HisTrap™ HP column (Cytiva, USA). Elution was performed with a linear gradient of imidazole (20 mM HEPES pH 7.5, 300 mM KCl, 800 mM imidazole, 10 mM β-mercaptoethanol, 10 % glycerol). Eluted fractions were analyzed by SDS-PAGE, and selected fractions were transferred into a dialysis membrane (Spectra/Por^®^4 Dialysis Membrane Standard RC Tubing MWCO: 12–14 kD, Spectrum Laboratories). The protein was dialyzed against 2 L dialysis buffer (20 mM HEPES pH 7.5, 100 mM KCl, 5 mM MgCl_2_, 1 mM DTT, 10 % glycerol) at 4 °C overnight to remove imidazole. The proteins were concentrated to 1.1–1.2 mg/ml (Amicon^®^ Ultra – 15 Regenerated Cellulose 30,000 NMWL; Merck, Germany), and filtrated using centrifugal filters (0.2 μm; VWR, USA) at 8,000 rpm and 4 °C for 3 min. Purified proteins were frozen in liquid nitrogen and stored at −80 °C.

### Helicase assay

Helicase activities of both SmeIF4A isoforms were determined by helicase assays, as previously described^15^. In brief, 1 µM of a 10mer RNA substrate modified with Cyanine 3 (10mer-Cy3; 5’-[Cy3] GCU UUC CGG U-3’; Microsynth AG, Germany) was annealed with a 16mer RNA substrate modified with Black Hole Quencher2 (16mer-BHQ2; 5’-ACU AGC ACC GGA AAG C [BHQ2]−3’; Microsynth AG, Germany) in duplex RNA buffer (25 mM HEPES-KOH pH 7.4) at 80 °C for 5 min to generate dsRNA. Next, the reaction was incubated at RT for 1 h and placed on ice for 10 min. A 10-fold excess (10 µM) of 10mer competitor RNA (10mer-competitor; 5’-GCU UUC CGG U-3’; Microsynth AG, Germany) was added, which captures released quencher RNA, and incubated on ice for 10 min. The maximum fluorescence signal was determined by the single-stranded (ss) 10mer-Cy3, which had not been annealed to the 16mer-BHQ2 before. The helicase reaction consisted of: 3 mM ATP pH 7.4, 100 nM dsRNA or ssRNA, and 6.2 µM SmeIF4A-a or 7.0 µM SmeIF4A-b, respectively, in assay buffer (150 mM HEPES-KOH pH 7.4, 15 mM Mg(CH_3_COO)_2_, 10 mM DTT, 500 mM CH_3_CO_2_K). Two negative controls with either no protein or no ATP were included. The reaction was prepared in black flat-bottom 96-well plates (Thermo Fisher Scientific, USA), and the protein added last, just before transferring the plate to the Infinite M Plex plate reader (TECAN, Germany) to start the measurements. Samples were excited at 540 nm, and fluorescence signals measured at 575 nm. In total, 1,000 kinetic cycles were performed at 25 °C with an interval time of 14 s.

### Thermal Shift Assay (TSA)

TSA experiments were performed by incubating 18.6 µM SmeIF4A-a or 20.9 µM SmeIF4A-b, respectively, each with 1 mM AMP-PNP (Roche, Switzerland), 50 µM poly-purine RNA (AG)_5_ (Biomers, Germany), and 100 µM of eIF4A inhibitors (rocaglates or pateamines) in TSA buffer (20 mM HEPES pH 7.5, 100 mM KCl, 5 mM MgCl_2_, 1 mM DTT, 10 % (v/v) glycerol) for 10 min on ice for complex formation. Next, 19.7 µl of this reaction were pipetted in MicroAmp™ Fast Optical 96-well plates (Applied Biosystems, USA), and 75 µM SYPRO™ Orange (Invitrogen, USA) were added. The 96-well plate was covered with an adhesive film (Applied Biosystems, USA) and transferred to a real-time PCR device (QuantStudio™ 3; Applied Biosystems, USA), which was controlled with the QuantStudio™ Design & Analysis software (v 1.5.2; Applied Biosystems, USA). The protein sample was exposed to a heating rate of 1.6 °C/s until 10 °C were reached and kept constant for 2 min. Next, the temperature was stepwise increased (0.05 °C/s) to 95 °C and kept constant for 1 min. Finally, the temperature was decreased by 1.6 °C/s until 10 °C were reached and kept for 1 min. Protein samples were excited at 472 nm, and emission was measured at 570 nm according to the spectroscopic maxima of SYPRO™ Orange. Resulting melting curves were analyzed using the Protein Thermal Shift software (v 1.3; Thermo Fisher Scientific, USA).

### Ethics statement

All animal experiments performed at the Justus Liebig University Giessen were conducted in accordance with the European Convention for the protection of vertebrate animals used for Experimental and other Scientific Purposes (ETS, No. 123; revised Appendix A), and have been approved by the Regional Council Giessen, Germany (V54-19c 20 15 h 01 GI18/10).

### Laboratory cycle of *S. mansoni*

The life cycle of *S. mansoni* was maintained by infecting *B. glabrata* snails as intermediate host, and the Syrian hamster *Mesocricetus auratus* as final host. Polymiracidial infection of the intermediate host was performed using a Liberian strain of *S. mansoni*^86^. Cercarial shedding was induced by exposing infected snails to a light source 28 days post infection (dpi). Cercariae were collected to infect hamsters as the final host. From these, adult worms were recovered by hepatoportal perfusion 46 dpi. The worms were transferred into petri dishes filled with pre-warmed (37 °C) M199 medium (Gibco, Germany) supplemented with 10 %(v/v) newborn calf serum (Sigma-Aldrich, US), 10 mM HEPES pH 7.4 (Carl Roth, Germany), and 1 %(v/v) antibiotic-antimycotic solution (ABAM; CCPro, Germany) containing 10,000 U penicillin, 10 mg streptomycin, and 25 µg amphotericin B per ml (M199 3+). The parasites were incubated at 37 °C and 5 % CO_2_ until further use.

### Inhibitor treatment of adult *S. mansoni in vitro*

*S. mansoni* worms collected on the day of perfusion were incubated for 24 h at 37 °C and 5 % CO_2_ to select only stable couples for inhibitor treatments. The *in vitro* culture was prepared by transferring 15 *S. mansoni* couples, randomly selected from different hamsters, into individual wells of 6-well culture plates, each filled with 5 ml pre-warmed M199 3 + medium using feather-weight tweezers. For each approach, three wells of a 6-well plate were prepared as technical replicates. Each experiment was conducted at least three times (*n* = 3). A total of five compounds from the rocaglate class and two pateamines were tested on adult *S. mansoni in vitro*. The compounds were added to each well at a dilution of 1:1,000. Control worms were incubated with 0.1 % DMSO (corresponding amount as in the treatment group). The worms were daily transferred to fresh medium in a 6-well plate, and the compounds were added over a period of 7 d. The culture plates containing 1–3 d old eggs laid *in vitro* were used for hatching assays.

For wo experiments, five *S. mansoni* couples of individual hamsters, were transferred into each well of a 12-well plate filled with M199 3 + medium using feather-weight tweezers. Except for the zotatifin treatment (*n* = 3), each approach was repeated six times (*n* = 6). Couples were either treated with the rocaglates zotatifin, RocA, and silvestrol, or the pateamines PatA, and DMDAPatA until day 7. Medium and compounds were refreshed daily until day 7. After this treatment period, medium was replaced every 2–3 d, and compounds were washed out until day 14. Worms were analyzed for different scoring parameters, as explained below. After 7 d of wo, two couples of each approach were incubated with EdU and subsequently used for an EdU assay and analyzed by CLSM, as described below. Remaining couples were incubated until day 14 and then also incubated with EdU to analyze stem-cell proliferation.

### Test compounds

The rocaglates CR-31-B (+), CR-31-B (–), zotatifin, and RocA were purchased from MedChemExpress (USA). Silvestrol was obtained from the Sarawak Biodiversity Centre (Kuching, Malaysia). PatA and DMDAPatA were kindly prepared and provided by the lab of Prof. A. Fürstner (Max-Planck-Institut für Kohlenforschung; Mühlheim an der Ruhr, Germany)^87^. All compounds were dissolved in DMSO and stored at −20 °C. The compounds were used at the following final concentrations: 3 nM PatA, 25 nM CR-31-B (+) and CR-31-B (–), 200 nM RocA, silvestrol, and DMDAPatA, and 700 nM zotatifin depending on previously determined CC_50_ values (Supplementary Table S2). CR-31-B contains a racemic mixture of two enantiomers: CR-31-B (+) and CR-31-B (–), while CR-31-B (+) is the biologically inactive enantiomer^7,52.^

### Scoring parameters

During the inhibitor treatment period, worms were daily inspected under an inverted microscope (DM IL LED; Leica Microsystems, Germany) for the following scoring parameters: (i) pairing stability, (ii) attachment to the cell culture dish, (iii) motility, and (iv) egg production. Worm motility was scored from 0 to 4 with 0 = no motility; 1 = minimal activity restricted to the gut, head or tail region; 2 = reduced motility; 3 = normal movement; 4 = hyperactivity as described elsewhere^88,89,90.^ Egg production was determined by counting the eggs laid *in vitro*.

### Hatching assay

Eggs laid *in vitro* by treated and control (DMSO-treated) worms were incubated with 20 % (v/v) NCS at 37 °C and 5 % CO_2_ for 7 d. ABAM was added every 2–3 d at 1 % (v/v). After 7 d, the medium was replaced by artificial tap water (3 mM CaCl_2_, 1 mM MgCl_2_, 43.42 mM K_2_CO_3_, 547.56 mM NaHCO_3_, 0.24 mM NaOH), and eggs were exposed to a light source at RT for 2 h. Prior counting, plates were incubated for at least 1 h at 4 °C to slow down movement of hatched miracidia.

### Lipid staining

After 7 d of treatment, vitellogenesis of treated and control worms was analyzed by staining the lipid droplets present in fully differentiated S3/S4 vitellocytes^62,63.^ To this end, a modified version of the Oil-Red O staining^64,65,^^65^ was applied as described before^45^. Two *S. mansoni* couples of each biological replicate were separated by incubation in 0.26 % (w/v) ethyl 3-aminobenzoate methanesulfonate (tricaine; Sigma-Aldrich, US) dissolved in M199 3+, as described elsewhere^40^. Separated females were fixed in 2 % paraformaldehyde (PFA) in PBSTx (1x PBS + 0.3 % Triton X-100; Sigma-Aldrich, US) at 4 °C for 24 h. The worms were rinsed twice in 1x PBSTx and incubated in 99 % propane-1,2-diol (Merck, Germany) on a rocker at RT for 5 min. Then, worms were stained in 0.5 % (w/v) Oil-Red O (Sigma-Aldrich, US) dissolved in propane-1,2-diol with moderate agitation at RT for 45 min. Next, worms were washed twice in 85 % propane-1,2-diol at RT for 5 min each before performing a final washing step in 1x PBS. Worms were embedded in ROTI Mount FluorCare (Carl Roth, Germany) on microscopic slides and immediately analyzed by phase contrast microscopy (DM IL LED; Leica Microsystem, Germany) using the WaveImage software (VWR, USA).

### Stem-cell proliferation assay and confocal laser scanning microscopy (CLSM)

Stem-cell proliferation was investigated after treating *S. mansoni* couples with eIF4A inhibitors for 7 d *in vitro*. For this, the thymidine analog EdU^66^ was added to three *S. mansoni* couples of each biological replicate at a final concentration of 10 µM (Thermo Fisher Scientific, USA) at 37 °C and 5 % CO_2_ for 24 h. For subsequent analysis, couples were separated by 0.26 %(w/v) tricaine dissolved in M199 3 +^40^ and male and female worms were separately fixed in 4% PFA/PBSTx at 4 °C overnight. The next day, worms were rinsed in PBSTx for 3 min and dehydrated in an ascending methanol (MeOH) series (50 % MeOH in PBSTx, 100 % MeOH) for 10 min each. Worms were finally stored in fresh 100 % MeOH at −20 °C until further use. For the EdU assay, the Click-iT Plus EdU Alexa Fluor 488 imaging kit (Thermo Fisher Scientific, USA) was used as described before^45^. Samples were rehydrated in 50 % MeOH in PBSTx, and PBSTx at RT for 10 min each. Worms were bleached under bright light in 1.2 % (v/v) H_2_O_2_, 5 % (v/v) formamide, 0.5x saline-sodium citrate (SSC) pH 7.0 in DEPC-treated water for 30 min. After bleaching, permeabilization was performed in 10 µg/ml proteinase K (Ambion, UK) in PBSTx for 25 min. Worms were post-fixed in 4 % PFA/PBSTx for 10 min followed by two washing steps in 1x PBS for 5 min each. Total DNA was stained by 10 µM Hoechst 33342 (Sigma-Aldrich, US) at 4 °C overnight. The parasites were washed three times in PBSTx for 10 min each and mounted on slides, as described before^91^.

Also for CLSM, we separated *S. mansoni* couples and investigated morphological changes in males and females. For this, the worms were fixed in an alcohol formalin acetic acid fixative (AFA) composed of 2 % acetic acid, 1.1 % PFA, and 66.7 % ethanol at RT for 24 h, as described before^45^. Staining occurred in Certistain carmine red (Merck, Germany) on a shaker at RT for 1 h. The parasites were destained several times in acidic ethanol, consisting of 70 %(v/v) ethanol and 2.5 %(v/v) hydrochloric acid, for 5 min each. Dehydration was performed with an ascending ethanol series (80 % 90 % 100 %) for 5 min each until worms were embedded on slides using Euparal.

EdU- and carmine-red stained worms were analyzed using an inverted CLSM (TCS SP5 VIS) and the LAS AF software (Leica Microsystems, Germany). The specimen were excited with an argon laser at 488 nm, while Hoechst 33342 was excited at 405 nm. Background signals and optical section thickness were defined by setting the pinhole size to airy unit 1^92,93^. Proliferating cells were quantified by counting EdU^+^ in the immature part of the ovary (containing stem cell-like oogonia) and in the testis (spermatogonia) in the maximum plane extension of these organs. The area of the organs was determined using the selection brush tool of ImageJ^94,95,96.^ The number of EdU^+^ cells per mm^2^ was calculated as described previously^61^.

### Statistical analyses

Statistical analyses were performed using the GraphPad Prism V.8 software (GraphPad Software; San Diego, USA) applying the two-tailed *t*-test for normally distributed data or the two-tailed Mann-Whitney test for non-normally distributed data. Normality was determined by the Shapiro-Wilk test. Statistical differences with *p* < 0.05 were considered significant.

## Supplementary Information

Below is the link to the electronic supplementary material.

Supplementary Material 1

## Data Availability

Data associated with this study have not been deposited in any publicly available repositories. All relevant data are included in the article or in the supplement.
