# Supplementary material for: Rocaglates and pateamines target the DEAD-box RNA helicase eIF4A from Schistosoma mansoni and demonstrate anti-schistosomal activity in vitro
Source: Sci Rep. 2026 Aug 1;16:23723. doi: 10.1038/s41598-026-63203-w (PMC13428745; doi:10.1038/s41598-026-63203-w)
Supplement: Supplementary file 1 — Supplementary Material 1 [file 41598_2026_63203_MOESM1_ESM.docx]

**Supplementary information**

Rocaglates and pateamines target the DEAD-box RNA helicase eIF4A from *Schistosoma mansoni* and demonstrate anti-schistosomal activity *in vitro*

Sophie Welsch^1^*, Francesca Magari^2^, Annika S. Mokosch^3^, Simone Haeberlein^1^, Stefanie Gerbig^4^, Bernhard Spengler^3^, Arnold Grünweller^2^*, and Christoph G. Grevelding^1^*

^1^ Biomedical Research Center Seltersberg (BFS), Institute of Parasitology, Justus Liebig University Giessen, 35392 Giessen, Germany

^2^ Institute of Pharmaceutical Chemistry, Philipps University Marburg, 35037 Marburg, Germany

^3^ Institute of Inorganic and Analytical Chemistry, Justus Liebig University Giessen, 35392 Giessen, Germany

^4^ TransMIT Gesellschaft für Technologietransfer mbH, 35394 Giessen, Germany

**Supplementary materials and methods**

***In vitro* culture and zotatifin treatment of *S. mansoni* couples**

For AP-SMALDI MSI experiments, ten *S. mansoni* couples, mixed from different hamsters, were cultured per well in a 6-well plate filled with M199 3+ medium. Couples were treated daily with 700 nM zotatifin and incubated at 37 °C and 5 % CO_2_ until d 7. Control worms were incubated with 0.1 % DMSO (corresponding amount as in the treatment group). Four wells were prepared for each approach, and the worms of each well were fixed for AP-SMALDI MSI (see below) at different time points (after 1, 3, 5, and 7 d).

**Fixation of *S. mansoni* for AP-SMALDI MSI**

After *in vitro* treatment of *S. mansoni* couples with zotatifin, worms were fixed for AP-SMALDI MSI at different time points (1, 3, 5, 7 d) following a previous protocol^1^. For this, worms were briefly transferred into PBS pH 7.4 using feather-weight tweezers to remove excess drug and medium. Next, worms were placed in 50 µl of a 6.6 % glutaraldehyde solution (grade I, 25 % in H_2_O; Sigma-Aldrich GmbH, St. Louis, US) in PBS on a glass slide, frozen in liquid nitrogen, and stored at -80 °C until further use.

**Sectioning of *S. mansoni***

Sectioning of worms was carried out on a cryostat HM525 (Epredia Dreieich, Germany). The sectioning protocol is based on Kadesch et al.^2^. Aqueous gelatin solution with a mass concentration of *β* = 80 g/L (water: LC-MS grade, VWR International GmbH, Darmstadt, Germany; gelatin: pharm. Eur., VWR, Radnor, US) was prepared. 15 µL gelatin solution were spotted on a stainless-steel sample holder (*d* = 6 mm) and frozen at –25 °C in the cryotome for 30 min. The top part of the droplet was sectioned afterwards to yield a flat plateau. Fixed and frozen worms were thawed in a desiccator at 22 °C for 30 min before transferring them with feather-weight tweezers in 200 µL water for 30 s to rinse off residual fixative. Subsequently, they were placed on the prepared gelatin surface and mounted in the cryotome. The worms were covered with additional 15 µL of gelatin solution, then frozen at –25 °C in the cryotome for 30 min. Subsequently, the samples were cut into sections with a thickness of 20 µm. Optical images were recorded using a digital light microscope (VHX 5000, Keyence, Osaka, Japan) for quality control. Sections were stored at –80 °C until further use.

**AP-SMALDI MSI measurements**

Sections were taken out of the freezer and thawed in a desiccator at 22 °C for 30 min. They were pneumatically covered with a matrix solution consisting of 2,5-dihydroxybenzoic acid (DHB, Sigma-Aldrich GmbH, St. Louis, US) with a concentration of *β* (DHB) = 30 mg/mL. The solution was prepared by dissolving 2,5-DHB in 1:1 (v/v) acetone:water, followed by addition of pure trifluoroacetic acid to obtain a 0.1 % acidic solution (acetone, LiChrosolv, Merck, Darmstadt, Germany; TFA, spectroscopy grade, AppliChem GmbH Darmstadt, Germany). Matrix application was carried out using a SMALDIPrep sprayer (TransMIT GmbH, Giessen, Germany). We applied 140 µL of matrix solution to each sample with a flow rate of 10 µL/min at a nitrogen pressure of 1 bar.

An autofocusing AP-SMALDI5 AF ion source^3,4^ (TransMIT GmbH, Giessen, Germany) was coupled to a Q Exactive HF orbital trapping mass spectrometer (Thermo Fisher Scientific, Bremen, Germany) for MSI analyses. Laser settings were adjusted to 50 UV-laser pulses per pixel at a frequency of 100 Hz. Pixel sizes between 5 and 7 μm were set. Pixel sizes were chosen according to the available measurement time and sample size. The autofocusing feature ensuring laser operation with optimal focus was used for all measurements. The *m/z* range was 250 to 1,000 u. All measurements were performed in positive-ion mode with a mass resolution of 240,000 at *m/z* 200. Internal calibration was performed using a lock mass at *m/z* 716.12461, corresponding to [5DHB – 4H_2_O + NH_4_]^+5^. An acceleration voltage of 3.0 kV was set. The ion injection time was set to 500 ms, the s-lens level was set to 100 arbitrary units, and the capillary temperature was chosen to be 250 °C.

**Data analysis**

The Q Exactive HF mass spectrometer was run with the software Q Exactive Tune (version 2.4, Thermo Fisher Scientific, Bremen, Germany). The program “SMALDI Control” (V1.1-118, TransMIT GmbH, Giessen, Germany) was used to control the stage for image acquisition and the autofocus. XCalibur (Thermo Fisher Scientific, Bremen, Germany) was utilized to display mass spectra. Mirion software package was used for data visualization and image generation^6^. Bin width of the histogram was set to 0.004 u and the absolute mass variance of spectra was set to 0.005 u. Total ion current (TIC) normalization was carried out for each *m/z* signal for image generation. Chemical structures were drawn using ACD/ChemSketch (Advanced Chemistry Development Inc., Toronto, Canada). Lipid assignment was performed using lipid maps^7^ and metaspace (https://metaspace2020.eu)^8^.

**TUNEL assay**

After *in vitro* culture of *S. mansoni* couples treated with either rocaglates or pateamines for 7 d (see main manuscript), three couples of each approach and each biological replicate (n = 3) were separated by 0.26 % (w/v) tricaine dissolved in M199 3+^9^. Males and females were fixed in 4 % PFA/PBSTx at 4 °C overnight. Worms were dehydrated in an ascending methanol series and stored at -20 °C as described before for the EdU assay (see main manuscript). TUNEL labeling of apoptotic cells was carried out according to Wendt et al.^10^ with slight modifications. In brief, parasites were rehydrated in 50 % MeOH/PBSTx and PBSTx for 10 min each. Worms were bleached under bright light for 1 h, rinsed in PBSTx, and permeabilized with 20 µg/ml proteinase K (Ambion, UK) in PBSTx for 25 min. Next, worms were rinsed in PBSTx and post-fixed in 4 % PFA/PBSTx for 10 min. Parasites were then rinsed in PBS for 5 min each and incubated in terminal deoxynucleotidyl transferase (TdT) reaction buffer from the Click-iT^TM^ Plus TUNEL Assay for In Situ Apoptosis Detection kit (C10619; Thermo Fisher Scientific, US) for 1 h at 37 °C. Worms were incubated in the TdT reaction mix for 1 h at 37 °C, and subsequently stained by the Click-iT Plus TUNEL reaction mix for 30 min at 37 °C according to the manufacturer’s instructions. Parasites were rinsed in PBS for 5 min each, and total DNA was stained by 10 µM Hoechst 33342 at 4 °C overnight. Worms were washed three times in PBSTx for 10 min each, and mounted on slides as described elsewhere^11^.

Fluorescently labeled worms were analyzed using a CLSM (TCS SP5 VIS; Leica Microsystems, Germany). Parasites were excited with a HeNe 633 laser at 650 nm, while Hoechst 33342 was excited at 405 nm.


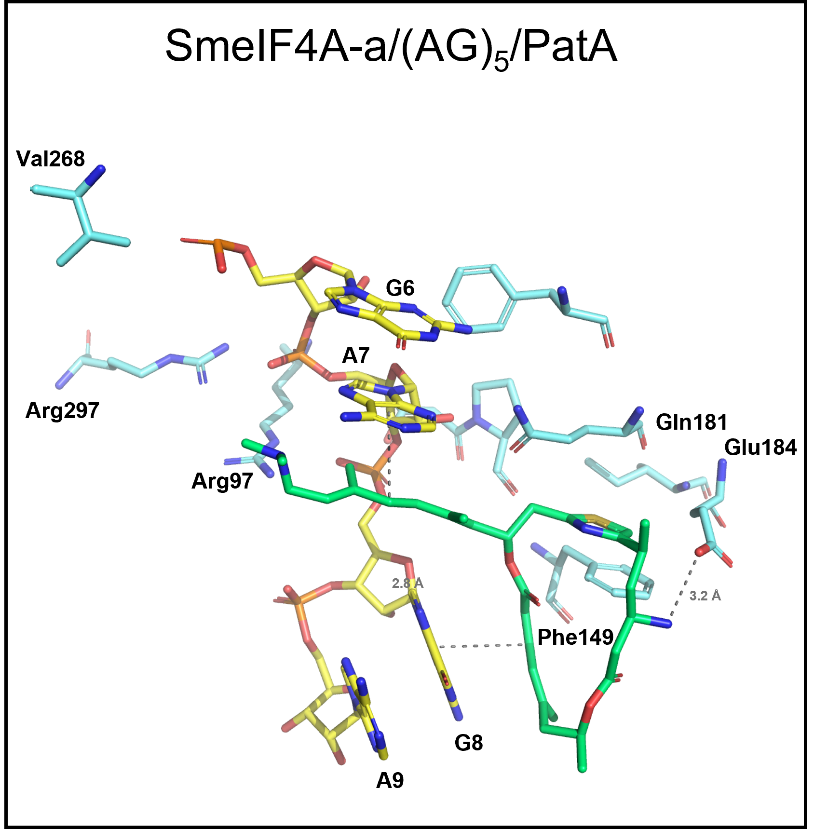


Supplementary Figure S1: Docking pose of SmeIF4A-a in complex with polypurine (AG)_5_ RNA, and the inhibitor PatA. The eIF4A inhibitor PatA (green stick model) in complex with the polypurine RNA (AG)_5_ (yellow sticks with phosphorus atoms colored orange) binds in the RNA binding pocket of SmeIF4A-a (cyan stick - nitrogen atoms in blue, oxygen atoms in red) in a similar fashion to human eIF4A^12^. PatA forms π-π stacking interactions with the RNA A7 and G8, and it is involved in a hydrogen bond with Glu184 (gray dashes).


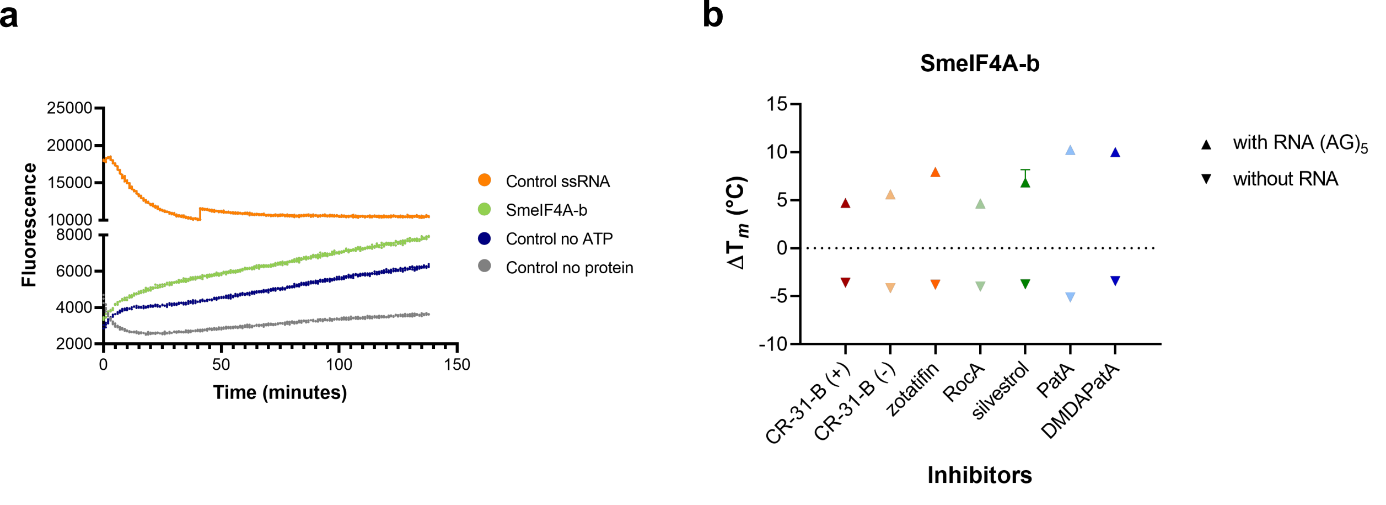


Supplementary Figure S2: Helicase assay of SmeIF4A-b and thermal denaturation measurements in complex with RNA and eIF4A inhibitors. (a) The helicase activity of SmeIF4A-b (green curve) is shown. Single-stranded RNA (orange curve) served as positive control and two negative controls were used: without protein (grey curve) and without ATP (blue curve). The latter unexpectedly showed an increase in fluorescent signals. (b) Plot of the ΔTm values after TSA with SmeIF4A-b in complex with (AG)_5_ RNA, AMP-PNP, and different rocaglates and pateamines (upward triangles). All compounds resulted in thermal shifts > 4 °C. Reactions without the addition of (AG)_5_ RNA (downward triangles) demonstrated a destabilizing effect with ΔTm values < 0 °C. Data are shown as mean of a triplicate ± SEM.


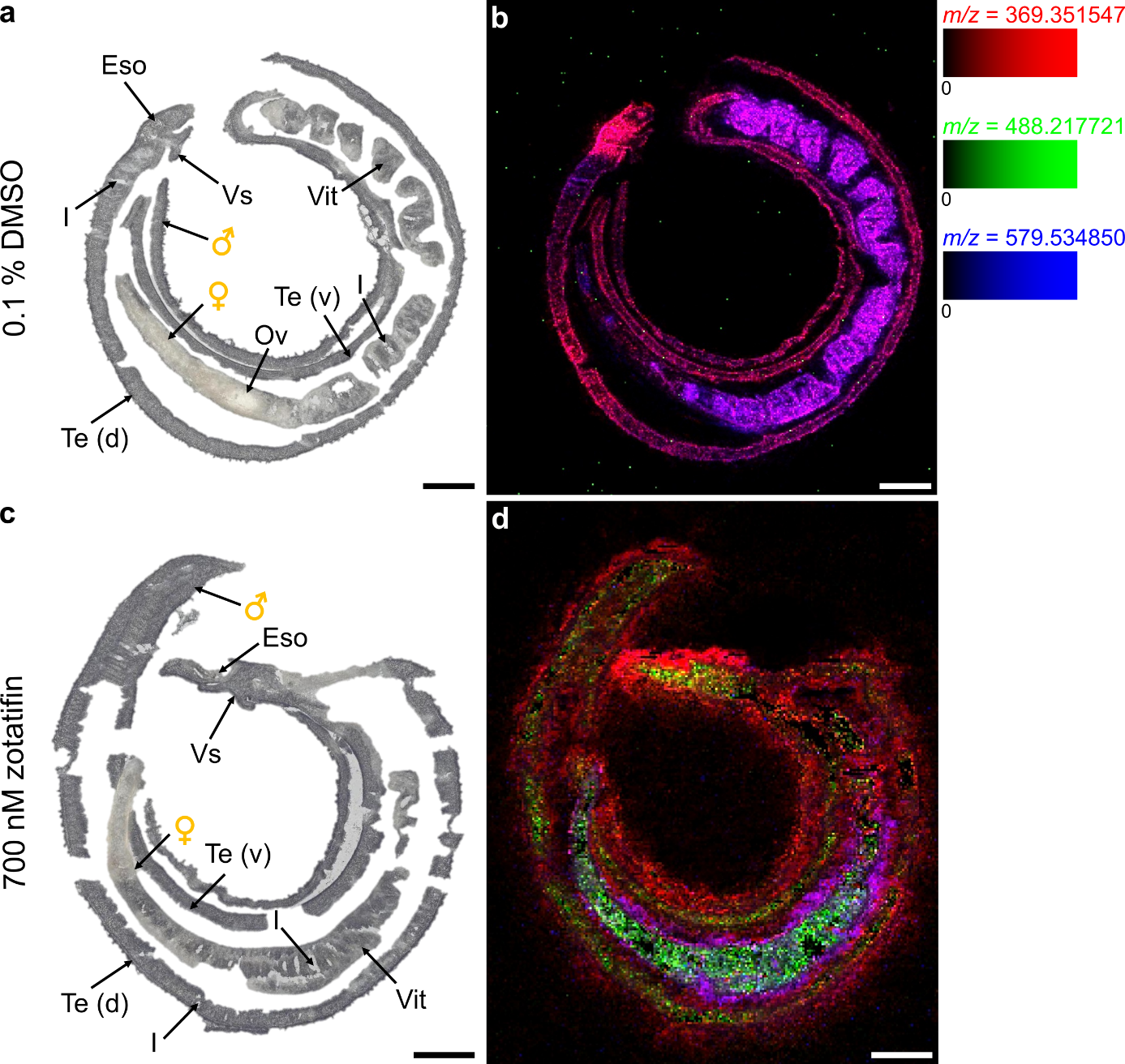


Supplementary Figure S3: Zotatifin was orally taken up by adult *S. mansoni*. (a) Optical image of a cryosection of a control *S. mansoni* couple (incubated with 0.1 % DMSO). (b) Representative MSI image of a control couple. No zotatifin or interfering signals at the same *m/z* values were detected in these untreated controls. (c) Optical image of a cryosection of couples treated with 700 nM zotatifin for 1 d. (d) MSI image of a couple treated with zotatifin. Signals were observed in the esophagus and intestine of males and females, but were absent in the tegument. Depicted analytes were cholesterol [M – H_2_O + H]^+^ *m/z* 369.351576 (red), zotatifin [M + H]^+^ *m/z* 488.217998 (green), and DG (34:0) [M – H_2_O + H]^+^ *m/z* 579.534686 (blue). Two sections of each biological replicate (n = 3) were analyzed, of which one representative couple each is shown here. Scale bars: 200 µm. Eso = esophagus, I = intestine, Ov = ovary, Te (d) = tegument dorsal, Te (v) = tegument ventral, Vit = vitellarium, Vs = ventral sucker. Measured *m/z* values and errors: b 369.351547 (-0.08 ppm), 488.217721 (-0.57 ppm), 579.534850 (0.28 ppm) d 369.351590 (-0.50 ppm), 488.217865 (-0.47 ppm), 579.534791 (0.18 ppm).


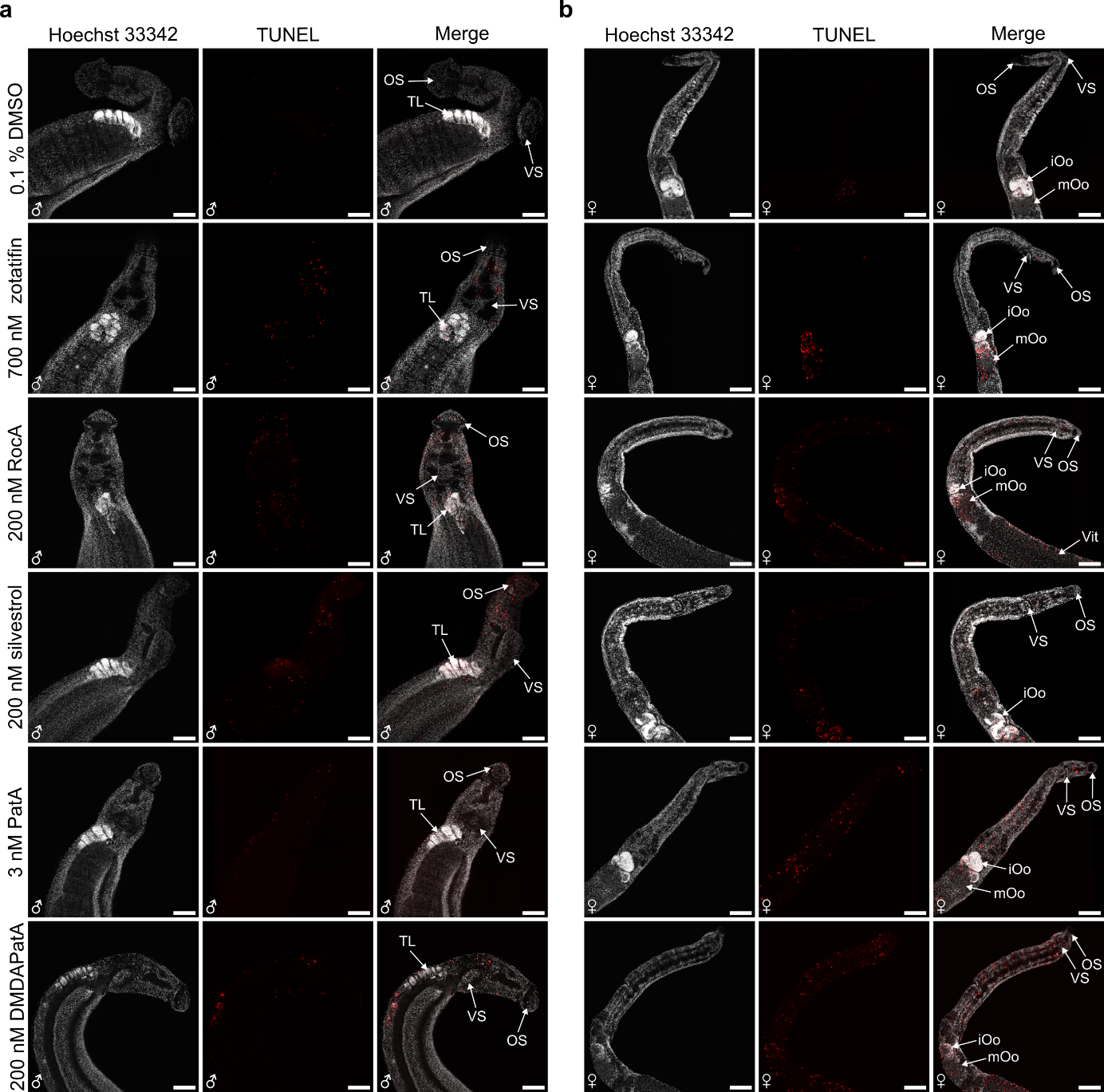


Supplementary Figure S4: Rocaglates and pateamines induced apoptosis in *S. mansoni* males (a) and females (b). *S. mansoni* couples were treated daily with the rocaglates zotatifin, RocA, and silvestrol or the pateamines PatA and DMDAPatA for 7 d *in vitro*. Then, couples were separated and single males and females were used for TUNEL assays to stain apoptotic cells (TUNEL^+^, red). Total DNA was visualized by Hoechst 33342 (grey). Few apoptotic cells were observed in control males and females (incubated with DMSO), which is a normal process in the parasite’s germline^10^. More TUNEL^+^ cells were observed after rocaglate and pateamine treatment, indicating induced apoptosis by these compounds. At least two worms were stained from each biological replicate (n = 3). Scale bars: 100 µm. iOo = oogonia, mOo = mature oocyte, OS = oral sucker, TL = testicular lobes, Vit = vitellarium, VS = ventral sucker.


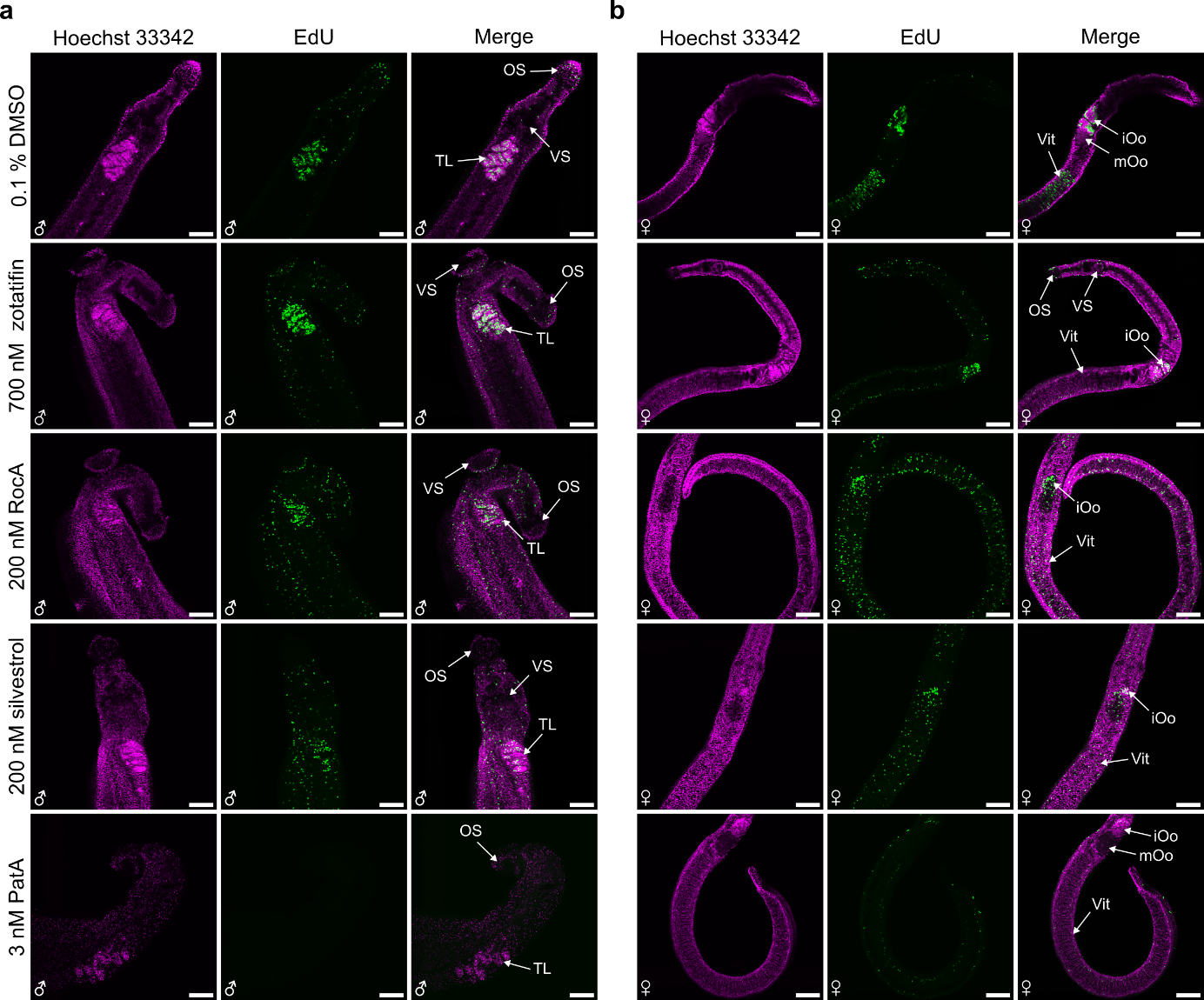


Supplementary Figure S5: A 7 d washout of rocaglate treatment partially recovered stem-cell proliferation in *S. mansoni* males (a) and females (b). *S. mansoni* couples were treated daily with the rocaglates zotatifin, RocA, and silvestrol or the pateamines PatA and DMDAPatA for 7 d *in vitro*. Afterwards, worms were cultured for 14 d without any compounds, and the medium was replaced every 2 - 3 d. After 7 d of washout, two couples of each approach were used for EdU assays and analyzed by CLSM. Total DNA was stained by Hoechst 33342 (magenta). EdU signals (green) were observed in males and females of the rocaglate washout groups, while no EdU^+^ cells were detected in PatA-treated males, and to a low extent in females. Scale bars: 100 µm. iOo = oogonia, mOo = mature oocyte, OS = oral sucker, TL = testicular lobes, Vit = vitellarium, VS = ventral sucker.


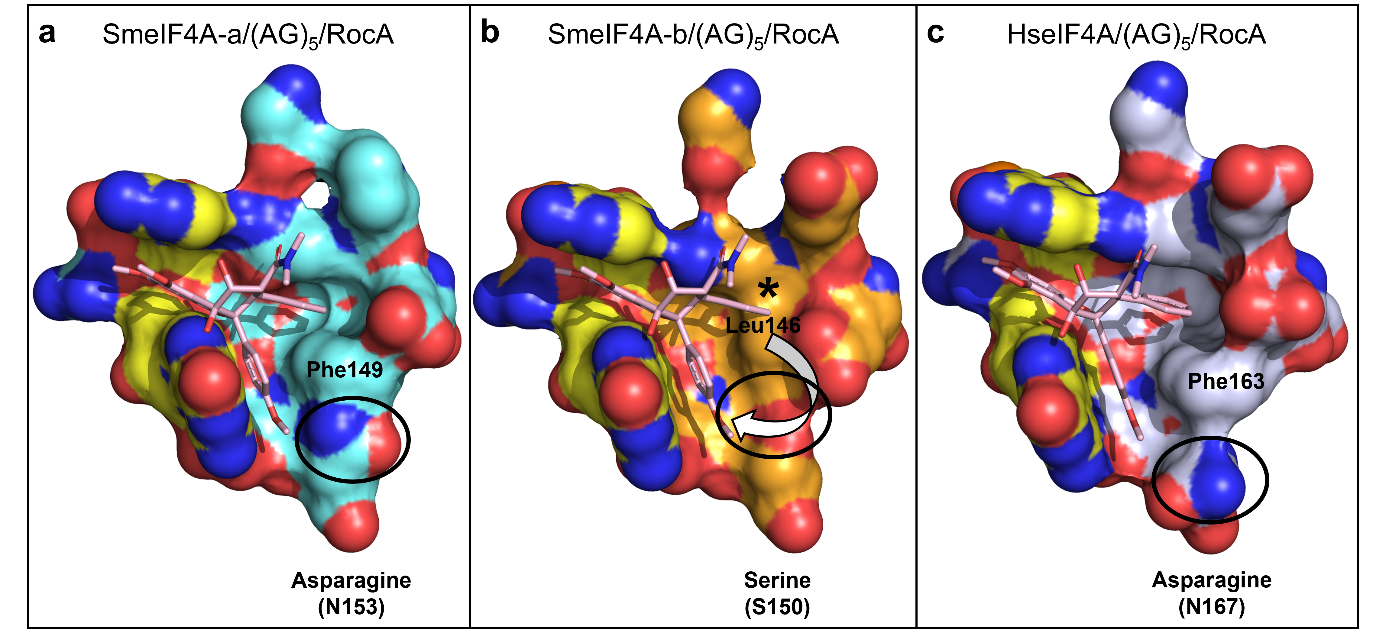


Supplementary Figure S6: Surface representation of SmeIF4A-a (a), SmeIF4A-b (b), and human eIF4A (c) in complex with polypurine (AG)_5_ RNA and the inhibitor RocA.RocA perfectly accommodates in the RNA-binding pocket of SmeIF4A-a (a) in a similar manner as in the human one (c). A Phe residue in SmeIF4A-a (a, Phe149) and in the human eIF4A (c, Phe163) is replaced by a Leu residue in SmeIF4A-b (b, Leu146; asterisk). In the human and SmeIF4A-a isoform, an Asn residue (c, N167 and a, N153; circles) closes the RNA pocket, whereas in the SmeIF4A-b isoform, the corresponding Ser residue (S150; circle) renders the pocket broader (arrow). This may promote a conformational rearrangement and better accommodation of RocA in the RNA binding pocket of SmeIF4A-b compared to the one of SmeIF4A-a (and human) eIF4A. Leu146 of SmeIF4A-b (b) may clash with ring C of RocA (represented as an asterisk in b), which nevertheless does not hamper the binding of RocA due to a higher freedom of movement in the broader RNA binding pocket.


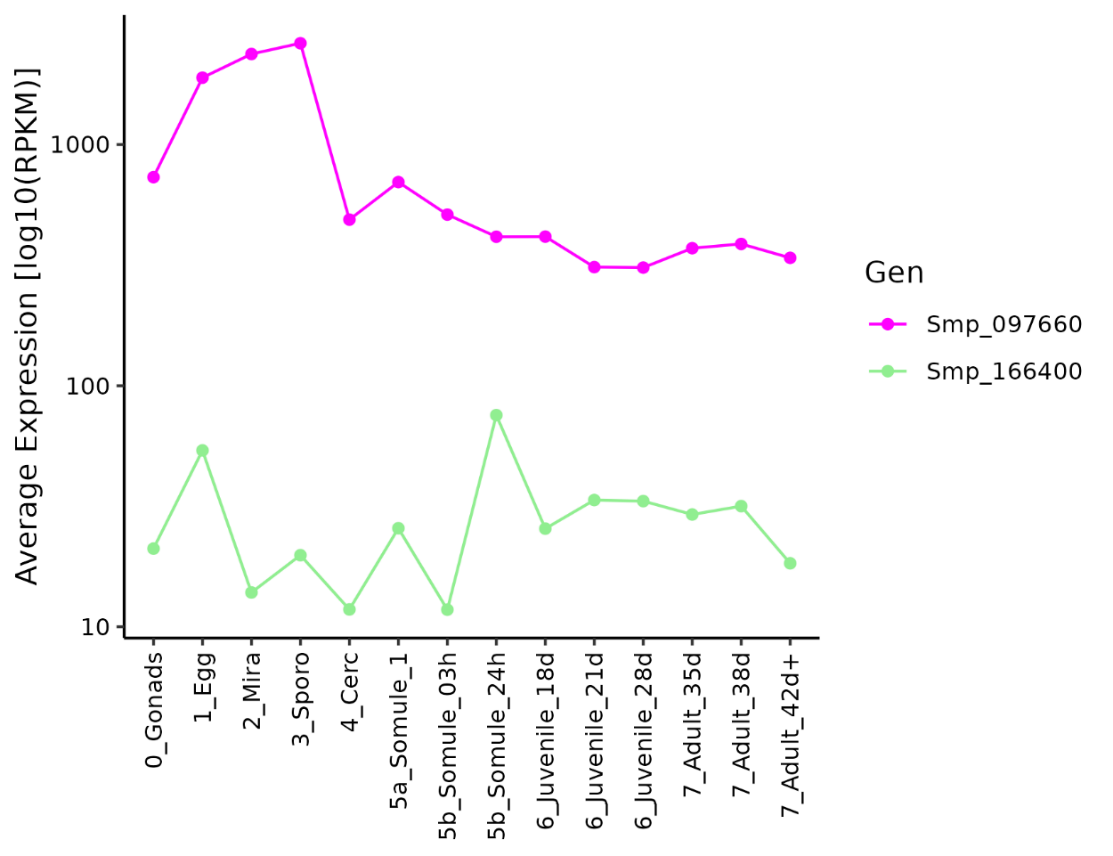


Supplementary Figure S7: Transcript levels of Sm*eif4a-a* and Sm*eif4a-b* in different life stages of *S. mansoni*. Previous bulk scRNA-seq^13^ determined transcript levels of Sm*eif4a-a* (Smp_097660, magenta) and Sm*eif4a-b* (Smp_166400, green) in all stages of the parasite’s life cycle. Sm*eif4a-a* is the predominant Sm*eif4a* isoform with higher expression levels in all life stages compared to Sm*eif4a-b*. The highest transcript levels of Sm*eif4a-a* are present in eggs, miracidia, and sporocysts. RPKM = reads per kilobase million.

Supplementary Table S1: Effects of rocaglates and pateamines on the melting temperatures (Tm) of SmeIF4A-a and SmeIF4A-b with or without RNA (AG)_5_. Differences in melting temperature (ΔTm) compared to the DMSO control and standard errors of the mean (SEM) are listed.

|  | **SmeIF4A-a (ΔTm ± SEM [°C])** | | **SmeIF4A-b (ΔTm ± SEM [°C])** | |
| --- | --- | --- | --- | --- |
|  | **with RNA** | **without RNA** | **with RNA** | **without RNA** |
| CR-31-B (+) | 0.28 ± 0.00 | -2.03 ± 0.06 | 4.71 ± 0.00 | -3.59 ± 0.05 |
| CR-31-B (-) | 5.12 ± 0.06 | -3.06 ± 0.06 | 5.62 ± 0.30 | -4.16 ± 0.18 |
| Zotatifin | 4.95 ± 0.06 | -3.06 ± 0.06 | 7.95 ± 0.40 | -3.81 ± 0.05 |
| RocA | 5.46 ± 0.06 | -2.94 ± 0.34 | 4.65 ± 0.25 | -3.99 ± 0.05 |
| Silvestrol | 7.19 ± 0.07 | -2.94 ± 0.00 | 6.84 ± 1.34 | -3.76 ± 0.05 |
| PatA | 6.31 ± 0.11 | -3.06 ± 0.30 | 10.23 ± 0.15 | -5.12 ± 0.05 |
| DMDAPatA | 6.31 ± 0.05 | -2.15 ± 0.25 | 10.00 ± 0.20 | -3.42 ± 0.05 |

Supplementary Table S2: Compounds used in this study and their previously published CC_50_ values.

| **Compound** | **Class** | **Origin** | **CC_50_** | **Cell type** | **Reference** |
| --- | --- | --- | --- | --- | --- |
| CR-31-B (+) | Rocaglates | Synthetic | - | - | - |
| CR-31-B (-) | Rocaglates | Synthetic | 2.6 nM | Monocytes | ^14^ |
| Zotatifin | Rocaglates | Synthetic | 78.1 nM | Monocytes | ^14^ |
| RocA | Rocaglates | Plant (*Aglaia sp.*) | - | - | - |
| Silvestrol | Rocaglates | Plant (*Aglaia sp.*) | 29 nM | Monocytes | ^14^ |
| PatA | Pateamines | Marine sponge (*M. hentscheli*) | 0.42 nM | HepG2 | ^12^ |
| DMDAPatA | Pateamines | Synthetic | 30.9 nM | HepG2 | ^12^ |

**References**

1. Mokosch, A. S., Gerbig, S., Grevelding, C. G., Haeberlein, S. & Spengler, B. High-resolution AP-SMALDI MSI as a tool for drug imaging in *Schistosoma mansoni*. *Anal. Bioanal. Chem.* **413**, 2755–2766 (2021).

2. Kadesch, P., Quack, T., Gerbig, S., Grevelding, C. G. & Spengler, B. Lipid topography in *Schistosoma mansoni* cryosections, revealed by microembedding and high-resolution atmospheric-pressure matrix-assisted laser desorption/ionization (MALDI) mass spectrometry imaging. *Anal. Chem.* **91**, 4520–4528 (2019).

3. Kompauer, M., Heiles, S. & Spengler, B. Autofocusing MALDI mass spectrometry imaging of tissue sections and 3D chemical topography of nonflat surfaces. *Nat. Methods* **14**, 1156–1158 (2017).

4. Kompauer, M., Heiles, S. & Spengler, B. Atmospheric pressure MALDI mass spectrometry imaging of tissues and cells at 1.4-μm lateral resolution. *Nat. Methods* **14**, 90–96 (2017).

5. Treu, A. & Römpp, A. Matrix ions as internal standard for high mass accuracy matrix-assisted laser desorption/ionization mass spectrometry imaging. *Rapid Communications in Mass Spectrometry* **35**, e9110 (2021).

6. Paschke, C. *et al.* Mirion--a software package for automatic processing of mass spectrometric images. *J. Am. Soc. Mass Spectrom.* **24**, 1296–1306 (2013).

7. Fahy, E., Sud, M., Cotter, D. & Subramaniam, S. LIPID MAPS online tools for lipid research. *Nucleic Acids Res.* **35**, W606–W612 (2007).

8. Palmer, A. *et al.* FDR-controlled metabolite annotation for high-resolution imaging mass spectrometry. *Nat. Methods* **14**, 57–60 (2017).

9. Collins, J. J. *et al.* Adult somatic stem cells in the human parasite *Schistosoma mansoni*. *Nature* **494**, 476–479 (2013).

10. Wendt, G. R. & James J Collins, I. Unusual inheritance of a functional cki homolog in the human pathogen *Schistosoma mansoni*. *Sci. Adv.* **11**, eaea4905 (2025).

11. Hahnel, S. *et al.* Gonad RNA-specific qRT-PCR analyses identify genes with potential functions in schistosome reproduction such as SmFz1 and SmFGFRs. *Front. Genet.* **0**, 170 (2014).

12. Magari, F. *et al.* Potent anti-coronaviral activity of pateamines and new insights into their mode of action. *Heliyon* **10**, (2024).

13. Lu, Z., Zhang, Y. & Berriman, M. A web portal for gene expression across all life stages of *Schistosoma mansoni*. *bioRxiv* https://doi.org/10.1101/308213 (2018) doi:10.1101/308213.

14. Obermann, W. *et al.* Rocaglates as antivirals: Comparing the effects on viral resistance, anti-coronaviral activity, RNA-clamping on eIF4A and immune cell toxicity. *Viruses* **14**, (2022).
